# Genome-wide identification of the opsin protein in *Leptosphaeria maculans* and comparison with other fungi (pathogens of *Brassica napus*)

**DOI:** 10.3389/fmicb.2023.1193892

**Published:** 2023-08-25

**Authors:** Marzieh Mohri, Ali Moghadam, Lenka Burketova, Pavel Ryšánek

**Affiliations:** ^1^Department of Plant Protection, Faculty of Agrobiology, Food, and Natural Resources, Czech University of Life Sciences, Prague, Czechia; ^2^Institute of Biotechnology, Shiraz University, Shiraz, Iran; ^3^Institute of Experimental Botany, Czech Academy of Sciences, Prague, Czechia

**Keywords:** G-protein-coupled receptors (GPCRs), G-proteins, opsin protein, *Leptosphaeria maculans*, *Brassica napus*

## Abstract

The largest family of transmembrane receptors are G-protein-coupled receptors (GPCRs). These receptors respond to perceived environmental signals and infect their host plants. Family A of the GPCR includes opsin. However, there is little known about the roles of GPCRs in phytopathogenic fungi. We studied opsin in *Leptosphaeria maculans*, an important pathogen of oilseed rape (*Brassica napus*) that causes blackleg disease, and compared it with six other fungal pathogens of oilseed rape. A phylogenetic tree analysis of 31 isoforms of the opsin protein showed six major groups and six subgroups. All three opsin isoforms of *L. maculans* are grouped in the same clade in the phylogenetic tree. Physicochemical analysis revealed that all studied opsin proteins are stable and hydrophobic. Subcellular localization revealed that most isoforms were localized in the endoplasmic reticulum membrane except for several isoforms in *Verticillium* species, which were localized in the mitochondrial membrane. Most isoforms comprise two conserved domains. One conserved motif was observed across all isoforms, consisting of the BACTERIAL_OPSIN_1 domain, which has been hypothesized to have an identical sensory function. Most studied isoforms showed seven transmembrane helices, except for one isoform of *V. longisporum* and four isoforms of *Fusarium oxysporum*. Tertiary structure prediction displayed a conformational change in four isoforms of *F. oxysporum* that presumed differences in binding to other proteins and sensing signals, thereby resulting in various pathogenicity strategies. Protein–protein interactions and binding site analyses demonstrated a variety of numbers of ligands and pockets across all isoforms, ranging between 0 and 13 ligands and 4 and 10 pockets. According to the phylogenetic analysis in this study and considerable physiochemically and structurally differences of opsin proteins among all studied fungi hypothesized that this protein acts in the pathogenicity, growth, sporulation, and mating of these fungi differently.

## 1. Introduction

Fungi must recognize signals from various plant cells and tissues at very early and later stages in order to respond to these signals and infect the host plant. Guanine nucleotide-binding proteins (G-proteins) convey the received information cell surface receptors. G-proteins are composed of three subunits: (α (38–52 *kDa*), β (35 *kDa*), and γ (8–10 *kDa*)) that come together to create heterotrimeric proteins. Intracellular signaling linked to G-proteins is triggered when an external ligand binds to the receptor. G-protein-coupled receptors (GPCRs) are considered the largest family of cell surface receptors in eukaryotes and prokaryotes and possess seven transmembrane (TM) domains. GPCRs are associated with intracellular G-proteins. The recognition of various signal molecules or ligands from host plants is carried out through the signaling from the fungal G-protein coupled receptor (GPCR). After perception of the signal by GPCRs, GDP changes to GTP at the α subunit of the G protein. Therefore, it results in the reduction/activation of target effectors (e.g., adenylate cyclase, protein kinases, ion channels, and phospholipases) by a conformational change of receptors (Wall et al., 1995; Kulkarni et al., 2005; Terakita, 2005; Brown et al., 2018; El-Defrawy and Hesham, 2020; Gao et al., 2021). GPCRs have been identified bioinformatically in various fungi, and they are divided into six families: the rhodopsin family (A), the secretin-receptor family (B), the metabotropic glutamate receptor family (C), fungal pheromone P- and α-factor receptors (D), fungal pheromone A- and M-factor receptors (E), and cyclic-AMP receptors from *Dictyostelium* (F). Opsin belongs to family A of the GPCR (rhodopsin family) (Nadarajah et al., 2018; El-Defrawy and Hesham, 2020).

Signal transduction by GPCRs enables fungi to coordinate cell transport, metabolism, and growth, which in turn promotes their survival, reproduction, and virulence. In fungi, several GPCR-regulated signaling pathways, such as the cAMP-activated Protein Kinase A (PKA) pathway, the mitogen-activated protein kinases (MAPK) cascades pathway, and the phospholipase C (PLC) pathway, affect cell growth, morphogenesis, mating, stress responses, metabolism, and pathogenicity (Gao et al., 2021). For example, one of the important plant hormones is Indole-3-acetic acid (IAA), involved in different responses such as growth and effects on Car0-like rhodopsins (they were early called Opsin-related proteins (OPRs)-like rhodopsins). It indicates the association of Car0-like rhodopsins with plant-fungus interactions (Lyu et al., 2016).

Rhodopsins have been found in a wide range of organisms, such as Eukarya, Bacteria, and Archaea. They consist of opsin apoproteins and a covalently linked retinal that absorbs photons to convert energy or stimulate intracellular/intercellular signaling. The functions of microbial rhodopsin are the conversion of the light to the electrochemical potential for the energy of the cell which is called light-driven ion pumps and also to regulate the cell processes by using light. The light-driven proton pumps are presumed to function in the generation of pH gradients or pH homeostasis. They might act as the acidification of some cell compartments that could be involved in the activation of certain biochemical responses (Waschuk et al., 2005; Idnurm et al., 2010). Animal rhodopsins play a variety of roles pertaining to vision and the sensation of light for nonvisual reasons such as circadian rhythms, sensing dawn/dusk, and body color change (Shichida and Matsuyama, 2009; Schmidt et al., 2011). Rhodopsins are divided into two groups: the first group is type II rhodopsins (animal rhodopsins), which consist of G-protein-coupled photoreceptors binding 11-*cis*-retinal and the auxiliary photoisomerases binding all-*trans*-retinal. The second group is type I rhodopsins (microbial rhodopsins), consisting of all-*trans*-retinal-binding transport and photosensory proteins of several organisms such as archaea, bacteria, and lower eukaryotes. Both type I and type II rhodopsins have the structure of seven TM α- helices consisting of an N-terminus outside the cell and a C-terminus inside the cell (Brown, 2004; Klare et al., 2008; Ernst et al., 2014).

Microbial opsins are transmembrane helix proteins in animals, archaea, fungi, and other eukaryotic microorganisms. These proteins belong to a class of retinal binding and play a role as light-responsive ion pumps or sensory receptors (Xue et al., 2008). Although the GPCRs play a role in several processes, such as pathogenicity, the genes have not been well studied in phytopathogenic fungi (Idnurm and Howlett, 2001; Terakita, 2005; Meena et al., 2018; Nadarajah et al., 2018). For example, there is little known about rhodopsins in one of the largest divisions of fungi, basidiomycetes. There are few studies regarding light responses in *Ustilago maydis* (a maize pathogen) belonging to basidiomycetes. There is a hypothesis that *Umops1* and *Umops2* are induced by white light in axenic cultures, and thus, the fungus synthesizes β-carotene (Estrada et al., 2009). Moreover, a few fungal opsins have been identified in *Neurospora crassa, Leptosphaeria maculans, Fusarium fujikuroi, Cryptococcus neoformans*, and *Botrytis cinerea*, but their functions are not yet known (Bieszke et al., 1999a; Idnurm and Howlett, 2001; Brown, 2004; Estrada and Avalos, 2009; Heller et al., 2012). Two GPCRs from class III in *Aspergillus* (GprC and GprD) are analogs to Gpr1 in *Saccharomyces cerevisiae* and have functions in sugar and oxylipin sensing. The deletion of these two genes (*gprC* or *gprD*) in *A. fumigatus* highlighted the fungus's inability to produce several toxic secondary metabolites. This deletion in turn affected the growth and pathogenicity in murine infections (Gehrke et al., 2010; de Souza et al., 2013). Another GPCR from class V (a common glucose and tryptophan sensor) is GprH, which has been found in several filamentous fungi and has a role in hyphal growth and sexual reproduction under carbon starvation in *A. nidulans* (Brown et al., 2015). The first opsin protein in fungi has been identified in *N. crassa* as Nop-1, an archaeal opsin homolog. The function of Nop-1 as rhodopsin in *N. crassa* has been revealed by gene and heterologous expression of this gene in *Pichia pastoris* (Bieszke et al., 1999a,b; Li et al., 2007). Transcriptomic analysis of *NOP-1* in wild-type and mutants has revealed that this gene acts in the late stage of conidiation. Its act is associated with the regulation of the expression of two genes involved in conidiation (*Al-2, NOC-10*, and *NOC-13*). Therefore, *NOP-1* is introduced as being involved in the modulation of carotenogenesis and repression of conidiation-specific gene expression in *N. crassa* (Bieszke et al., 2007). The study of the homolog gene *Sop1* in *Sclerotinia sclerotiorum* has revealed different functions of this gene, which is involved in the growth and virulence of *S. sclerotiorum* (Lyu et al., 2016).

The second most important oilseed crop is oilseed rape in the world. The global oilseed rape production is 31.8 million metric tons in 2022/2023.^1^ Many pests and diseases associated with *B. napus* include the crucifer flea beetle, cabbage stem flea beetle, clubroot, sclerotinia stem rot, and blackleg (Walker and Booth, 2001). Fungi represent the most serious pathogens in oilseed rape. Ascomycetes, *Leptosphaeria* spp., cause significant yield losses in all cultivation areas worldwide. *L. maculans* causes so-called blackleg disease, which is demonstrated by leaf and stem lesions (Fitt et al., 2006).

Few studies have comprehensively analyzed the structure and characteristics of microbial opsins in fungi. Microbial opsins are involved in responses to different environmental signals, such as sensory receptors. For example, light sensing influences fungal development, especially sporulation and mating (Idnurm and Heitman, 2005). Therefore, the identification of these proteins and understanding their functions in fungi could be useful in different areas of plant pathology and plant breeding to provide new resistance genotypes against these proteins. Therefore, this study aimed to identify the potential opsins in *L. maculans* and six other fungal pathogens of oilseed rape, as well as analyze their characters at the bioinformatics level that might have similar functions.

Our study provides a comprehensive analysis of opsin in *L. maculans* and six other fungi: *Alternaria alternata, S. sclerotiorum, B. cinerea, Verticillium dahliae, V. longisporum*, and *F. oxysporum*, which cause diseases in oilseed rape. To achieve this goal, we used bioinformatics pipelines for sequence alignment, domain structure classification, transmembrane helices analysis, subcellular location prediction, conserved domains and motifs, the prediction of secondary and tertiary structures, and protein-protein interactions. These results provide a crucial understanding of the opsin protein in seven fungal pathogens of oilseed rape and identify candidate genes for fungal pathogenicity, growth, and sporulation.

## 2. Results

### 2.1. Domain identification of opsin proteins from different fungi

**Figure 1:**
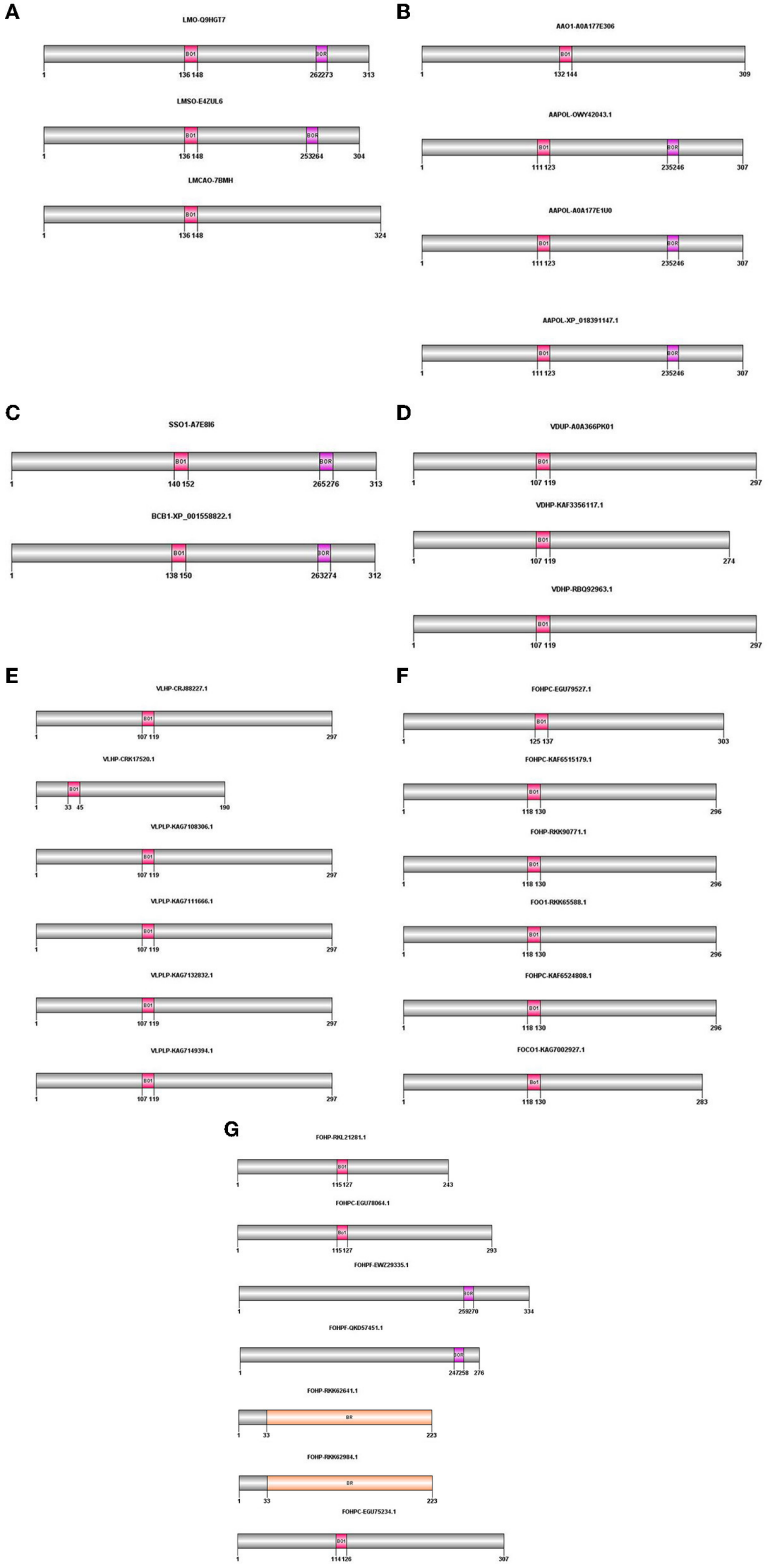
Domain architecture of opsin protein in all species using PROSITE, Pfam; The positions of these domains were drawn using Domain Graph (DOG) software. **(A)** *L. maculans*, **(B)** *A. alternata*, **(C)** *S. sclerotiorum, B. cinerea*, **(D)** *V. dahliae*, **(E)** *V. longisporum*, **(F, G)** *F. oxysporum*. BO1, BACTERIAL_OPSIN_1; BOR, BACTERIAL_OPSIN_RET; BR, Bac rhodopsin.

The proteins obtained from BLASTP were analyzed for the presence of conserved domains using the PROSITE database (https://prosite.expasy.org/scanprosite/). According to this database, bacterial rhodopsins were specifically detected, and they showed two patterns: the first pattern illustrated the isoforms containing BACTERIAL_OPSIN_1, and the second pattern indicated BACTERIAL_OPSIN_RET. Two of the above-mentioned patterns were found in two isoforms of *L. maculans* (LMO-Q9HGT7 and LMSO-E4ZUL6), three isoforms of *A. alternata* (AAPOL-OWY42043.1, AAPOL-A0A177E1U0, and AAPOL-XP_018391147.1), *S. sclerotiorum*, and *B. cinerea*. The BACTERIAL_OPSIN_1 domain was found in one isoform of *L. maculans* (LMCAO-7BMH), one isoform of *A. alternata* (AAO1-A0A177E306), all isoforms of *V. dahliae* and *V. longisporum*, and nine isoforms of *F. oxysporum* (FOO1-RKK65588.1, FOCO1-KAG7002927.1, FOHP-RKK90771.1, FOHP-RKL21281.1, FOHPC-EGU75234.1, FOHPC-EGU78064.1, FOHPC-EGU79527.1, FOHPC-KAF6515179.1, and FOHPC-KAF6524808.1). Two isoforms of *F. oxysporum* contain the BACTERIAL_OPSIN_RET domain (FOHPF-QKD57451.1 and FOHPF-EWZ29335.1). Two other isoforms of *F. oxysporum* (FOHP-RKK62641.1 and FOHP-RKK62984.1) showed that Bac rhodopsin conserved domain in the Pfam database (http://pfam.xfam.org/) (Figure 1).

### 2.2. Characterization of the opsin protein

Several physicochemical and biochemical parameters of the opsin protein in *L. maculans* and six other fungal species were calculated using the ProtParam tool of the ExPASy. They are listed in Table 1. These parameters indicated the variation of the length of the opsin protein from 190 aa (VLHP-CRK17520.1) to 334 aa (FOHPF-EWZ29335.1). The lowest molecular weight was also found in *V. longisporum* species (VLHP-CRK17520.1), at 21166.72 *kDa*, and the highest one in *F. oxysporum* (FOHPF-EWZ29335.1), at 36625.13 *kDa*. The protein charge stability was investigated using isoelectric points (pI) (Gasteiger et al., 2005). The range of isoelectric points varies from 5.54 in *B. cinerea* to 9.08 in *F. oxysporum*. The instability index value was calculated for the experimental evaluation of a protein's stability. When the instability index values are lower than 40, the protein is predicted to be stable (Guruprasad et al., 1990). The calculation of the instability index revealed that all proteins were stable experimentally. The aliphatic index of a protein is the relative volume that is occupied by aliphatic side chains (alanine, valine, isoleucine, and leucine) (Ikai, 1980; Gasteiger et al., 2005). Three isoforms of *A. alternata* (AAPOL-OWY42043.1, AAPOL-A0A177E1U0, and AAPOL-XP_018391147.1) have a lower aliphatic index, whereas other isoforms have the highest thermostability, ranging from 100.81 to 125.47. To calculate the grand average of hydropathicity (GRAVY) of a peptide or protein, the sum of hydropathy values of all of the amino acids was divided by the number of residues in the sequence (Kyte and Doolittle, 1982; Gasteiger et al., 2005). The GRAVY analysis revealed that all isoforms of the opsin protein were hydrophobic, with a GRAVY value above 0.

**Table 1:** Physicochemical parameters of opsin proteins of all isoforms using the ProtParam tool of the ExPASy.

**Name**	**Number of aa**	**Molecular weight**	**pI**	**Instability index**	**Aliphatic index**	**Gravy index**
LMO-Q9HGT7	313	34,273.93	5.88	22.37	114.38	0.506
LMSO-E4ZUL6	304	33,365.87	5.71	23.7	113.59	0.507
LMCAO-7BMH	324	35,649.51	6.25	21.89	110.49	0.408
AAO1-A0A177E306	309	33,753.27	5.62	26.28	105.79	0.442
AAPOL-OWY42043.1	307	34,417.76	6.22	25.58	94.72	0.25
AAPOL-A0A177E1U0	307	34,371.73	6.04	26.8	95.37	0.27
AAPOL-XP_018391147.1	307	34,371.73	6.04	26.8	95.37	0.27
SSO1-A7E8I6	313	34,485.83	5.74	29.86	105.69	0.438
BCB1-XP_001558822.1	312	34,323.01	5.54	23.97	114.39	0.548
VDUP-A0A366PK01	297	32,637.57	6.33	35.24	100.81	0.354
VDHP-KAF3356117.1	274	30,341.16	8.33	39.2	101.46	0.438
VDHP-RBQ92963.1	297	32,637.57	6.33	35.24	100.81	0.354
VLHP-CRJ88227.1	297	32,664.64	6.33	33.53	102.12	0.373
VLHP-CRK17520.1	190	21,166.72	5.56	34.22	117.47	0.675
VLPLP-KAG7108306.1	297	32,593.56	6.48	34.95	101.14	0.372
VLPLP-KAG7111666.1	297	32,621.57	6.33	35.24	101.14	0.363
VLPLP-KAG7132832.1	297	32,562.49	6.48	32.89	102.12	0.375
VLPLP-KAG7149394.1	297	32,531.48	6.48	32.89	102.12	0.381
FOO1-RKK65588.1	296	32,354.52	8.83	16.65	111.35	0.494
FOCO1-KAG7002927.1	283	30,868.78	9.08	17.06	108.55	0.456
FOHP-RKK62641.1	223	24,313.78	8.33	27.27	120.72	0.915
FOHP-RKK62984.1	223	24,353.07	9.06	30.68	122.91	0.987
FOHP-RKK90771.1	296	32,349.51	8.83	20.74	110.71	0.486
FOHP-RKL21281.1	243	26,344.3	6.53	15.55	101.11	0.4
FOHPF-EWZ29335.1	334	36,625.13	8.19	41.64	117.69	0.647
FOHPF-QKD57451.1	276	30,123.73	8.58	28.22	125.47	0.905
FOHPC-EGU75234.1	307	34,037.45	7.71	21.74	102.35	0.314
FOHPC-EGU78064.1	293	31,886.93	8.76	19.89	107.51	0.511
FOHPC-EGU79527.1	303	33,144.41	8.82	13.91	111.02	0.491
FOHPC-KAF6515179.1	296	32,384.59	8.83	15.12	112.67	0.512
FOHPC-KAF6524808.1	296	32,328.44	8.83	16.07	109.7	0.475

**Figure 2:**
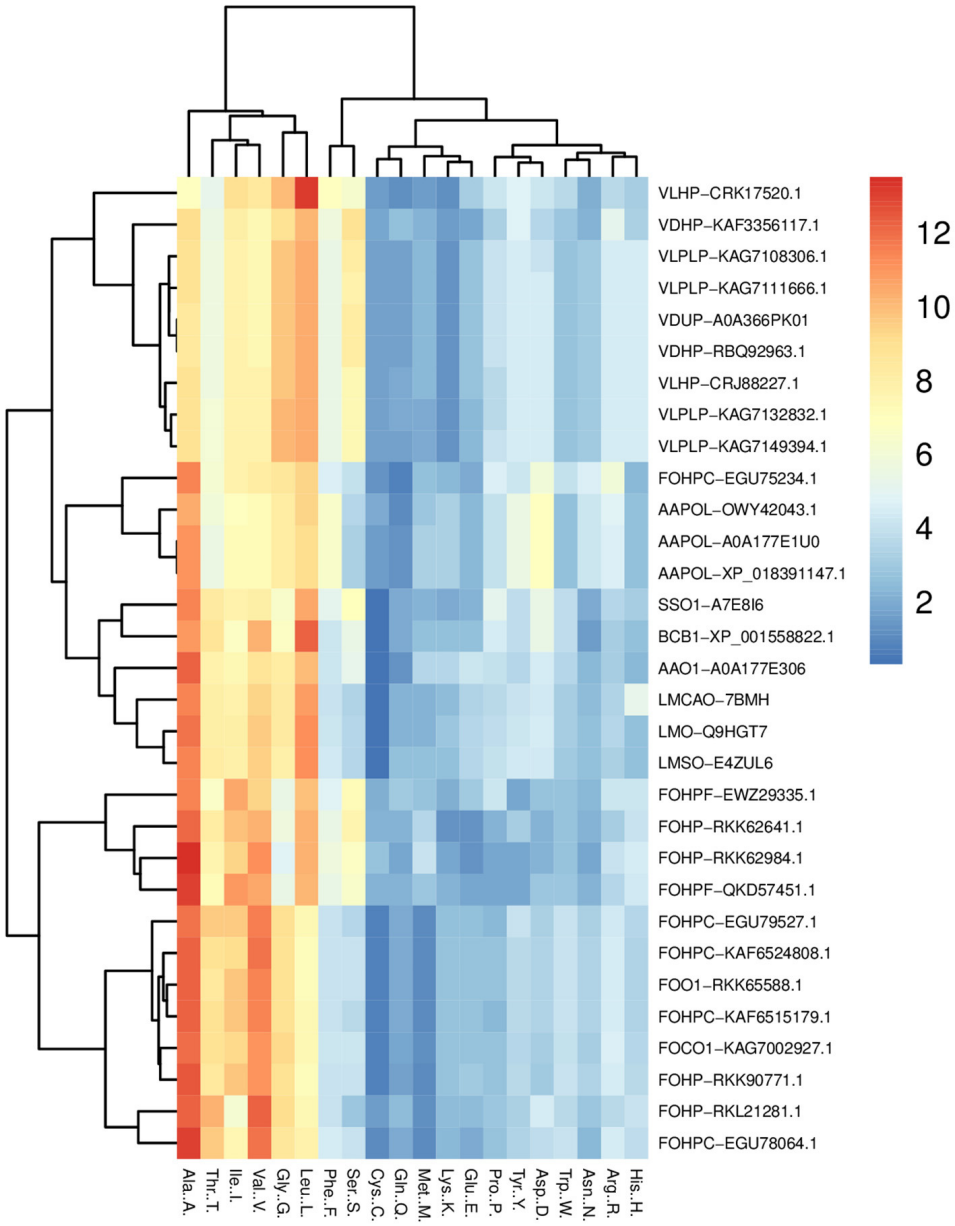
Heat map of the percentage comparison of the amino acids of opsin protein across all species (*L. maculans, A. alternata, S. sclerotiorum, B. cinerea, V. dahliae, V. longisporum*, and *F. oxysporum*) using R program. It illustrates the high percentage of six amino acids (leucine, glycine, valine, isoleucine, threonine, and alanine) in the opsin protein of these fungi.

The amino acid compositions of opsin proteins across species were compared using the heatmap package of the R program. This analysis shows that leucine, glycine, valine, isoleucine, threonine, and alanine have a greater portion of this protein than other amino acids (Figure 2). A comparison of the percentage amino acid composition of opsin proteins across all isoforms in terms of polar, nonpolar, positively and negatively charged amino acids showed that only one isoform of *A. alternata*, and all isoforms of *S. sclerotiorum, B. cinerea*, and *L. maculans* were enriched in nonpolar amino acids such as, alanine, valine and leucine, and polar amino acids such as, threonine. Three other isoforms of *A. alternata* have a high percentage of alanine, valine, and leucine from the non-polar amino acid group, along with aspartic acid from the negatively charged amino acid group.

The complex of two species of *Verticillium* (*V. dahliae* and *V. longisporum*) indicated the highest percentage of non-polar amino acids, including alanine, valine, leucine, glycine, and serine from the polar amino acid group. The percentage of amino acids in *F. oxysporum* is divided into three groups: the first group includes FOHPC-EGU79527.1, FOHPC-KAF6515179.1, FOHP-RKK90771.1, FOO1-RKK65588.1, FOHPC-KAF6524808.1, FOCO1-KAG7002927.1, FOHP-RKL21281.1, and FOHPC-EGU78064.1 and is rich in non-polar amino acids, including alanine, valine, isoleucine, glycine, and threonine from a polar group of amino acids. The second group of *F. oxysporum* (FOHPF-EWZ29335.1, FOHPF-QKD57451.1, FOHP-RKK62641.1, and FOHP-RKK62984.1) has a high percentage of non-polar amino acids, including alanine, valine, leucine, and isoleucine, and polar amino acids, including threonine and serine. The last isoform of *F. oxysporum* (FOHPC-EGU75234.1) presents a high percentage of alanine, valine, and leucine from non-polar amino acids, and threonine from polar amino acids, arginine as a positive amino acid, and aspartic acid as a negative amino acid (Supplementary Figure S1). The coevolutionary amino acid analysis revealed that tryptophan had been conserved among all species (Supplementary Figure S2).

### 2.3. Subcellular localization of the opsin protein in the different fungal pathogens of *B. napus*

Subcellular localization of the opsin protein of all isoforms was conducted using Loctree3 and illustrated that the majority of these proteins were localized in the endoplasmic reticulum membrane except for the two isoforms of *V. dahliae* (VDUP-A0A366PK01 and VDHP-RBQ92963.1) and five isoforms of *V. longisporum* (VLHP-CRJ88227.1, VLPLP-KAG7108306.1, VLPLP-KAG7111666.1, VLPLP-KAG7132832.1, and VLPLP-KAG7149394.1), which were localized in the mitochondrial membrane.

The other subcellular compartment is the nucleus. Proteins are synthesized in the cytoplasm, and they may be transported to the nucleus if they are needed there. There are binding sites on the protein sequence, known as nuclear-localized signals (NLSs), that mediate the protein's transportation to the nucleus (Brameier et al., 2007; Lu et al., 2021). Nuclear-localized signal analysis was conducted using the NucPred program. This analysis revealed that three isoforms of *A. alternata* (AAPOL-OWY42043.1, AAPOL-A0A177E1U0, and AAPOL-XP_018391147.1) and all isoforms of *Verticillium*, except for isoform VLHP-CRK17520.1, possessed NLS (Table 2). Transmembrane domain analysis was conducted using the PolyPhobius program. This analysis illustrates that most isoforms possess seven transmembrane domains, except for VLHP-CRK17520.1 with five TMs, FOHP-RKK62641.1, FOHP-RKK62984.1, and FOHP-RKL21281.1 that possess six TMs, and FOHPF-QKD57451.1 with eight TMs (Table 2; Supplementary Figure S3). This analysis shows that one sole isoform possesses a signal peptide (*B. cinerea)*. Three isoforms of *A. alternata* (AAPOL-OWY42043.1, AAPOL-A0A177E1U0, and AAPOL-XP_018391147.1), *S. sclerotiorum*, and all isoforms of *Verticillium*, except for two isoforms, VDHP-KAF3356117.1 and VLHP-CRK17520.1, possess N-glycan. Four isoforms of *F. oxysporum* (FOHPF-QKD57451.1, FOHPF-EWZ29335.1, FOHP-RKL21281.1, and FOHPC-EGU78064.1) possess N-glycan, and FOHPC-EGU75234.1 shows two sites of N-glycan in its sequence.

**Table 2:** Subcellular localization (using Loctree3), nuclear-localized signal prediction (using NucPred), and transmembrane helices analysis (using the PolyPhobius) of opsin protein of all species.

**Name**	**Subcellular localization**	**NucPred**	**Number of transmembrane helices**
LMO-Q9HGT7	Endoplasmic reticulum membrane	**-**	7
LMSO-E4ZUL6	Endoplasmic reticulum membrane	**-**	7
LMCAO-7BMH	Endoplasmic reticulum membrane	**-**	7
AAO1-A0A177E306	Endoplasmic reticulum membrane	**-**	7
AAPOL-OWY42043.1	Endoplasmic reticulum membrane	**NLS**	7
AAPOL-A0A177E1U0	Endoplasmic reticulum membrane	**NLS**	7
AAPOL-XP_018391147.1	Endoplasmic reticulum membrane	**NLS**	7
SSO1-A7E8I6	Endoplasmic reticulum membrane	**-**	7
BCB1-XP_001558822.1	Endoplasmic reticulum membrane	**-**	7
VDUP-A0A366PK01	Mitochondrion membrane	**NLS**	7
VDHP-KAF3356117.1	Endoplasmic reticulum membrane	**NLS**	7
VDHP-RBQ92963.1	Mitochondrion membrane	**NLS**	7
VLHP-CRJ88227.1	Mitochondrion membrane	**NLS**	7
VLHP-CRK17520.1	Endoplasmic reticulum membrane	**-**	5
VLPLP-KAG7108306.1	Mitochondrion membrane	**NLS**	7
VLPLP-KAG7111666.1	Mitochondrion membrane	**NLS**	7
VLPLP-KAG7132832.1	Mitochondrion membrane	**NLS**	7
VLPLP-KAG7149394.1	Mitochondrion membrane	**NLS**	7
FOO1-RKK65588.1	Endoplasmic reticulum membrane	**-**	7
FOCO1-KAG7002927.1	Endoplasmic reticulum membrane	**-**	7
FOHP-RKK62641.1	Endoplasmic reticulum membrane	**-**	6
FOHP-RKK62984.1	Endoplasmic reticulum membrane	**-**	6
FOHP-RKK90771.1	Endoplasmic reticulum membrane	**-**	7
FOHP-RKL21281.1	Endoplasmic reticulum membrane	**-**	6
FOHPF-EWZ29335.1	Endoplasmic reticulum membrane	**-**	7
FOHPF-QKD57451.1	Endoplasmic reticulum membrane	**-**	8
FOHPC-EGU75234.1	Endoplasmic reticulum membrane	**-**	7
FOHPC-EGU78064.1	Endoplasmic reticulum membrane	**-**	7
FOHPC-EGU79527.1	Endoplasmic reticulum membrane	**-**	7
FOHPC-KAF6515179.1	Endoplasmic reticulum membrane	**-**	7
FOHPC-KAF6524808.1	Endoplasmic reticulum membrane	**-**	7

### 2.4. Phylogenetic tree

**Figure 3:**
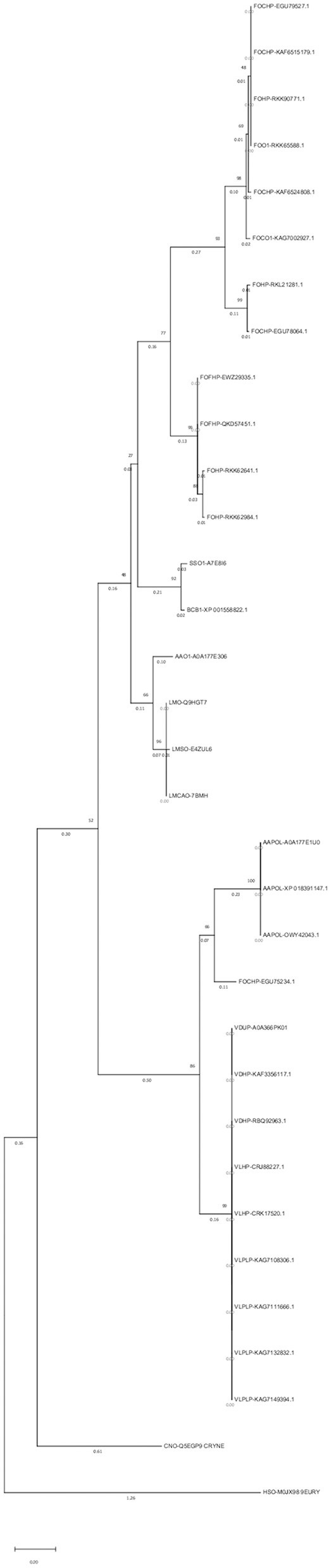
Phylogenetic tree using MEGAX. The phylogenetic tree illustrates the evolutionary relationship of opsin protein across all isoforms. Thirty-one isoforms of the opsin protein are clustered in six major groups and six subgroups.

An alignment file generated from multiple sequence alignment using the ClustalW tool in MEGAX, besides two outgroups, was used to construct a phylogenetic tree and analyze the evolutionary relationships of the opsin protein among different species (Figure 3). In the phylogenetic tree, 31 isoforms of the opsin protein from different fungal species were clustered into six major groups and six subgroups. Clades 1 and 2 included *F. oxysporum* and were divided into two subgroups. Two different species (*S. sclerotiorum* and *B. cinerea*) were grouped in the third clade. Clade 4 was divided into two subgroups: one subgroup consisted of one isoform of *A. alternata* (AAO1-A0A177E306), and three isoforms of *L. maculans* were grouped in the second subgroup of this clade. Clade 5 was also divided into two subgroups: the other isoforms of *A. alternata* were grouped in one subgroup of this clade, and one isoform of *F. oxysporum* (FOHPC-EGU75234.1) was in the other subgroup of this clade. Clade 6 consisted of a complex of two species of *Verticillium*: *V. dahliae* and *V. longisporum*.

A maximum parsimony tree was also created using the above-mentioned alignment file. The results showed identical main clades as the above-mentioned tree (Supplementary Figure S6).

### 2.5. Conserved motifs of opsin homologs

**Figure 4:**
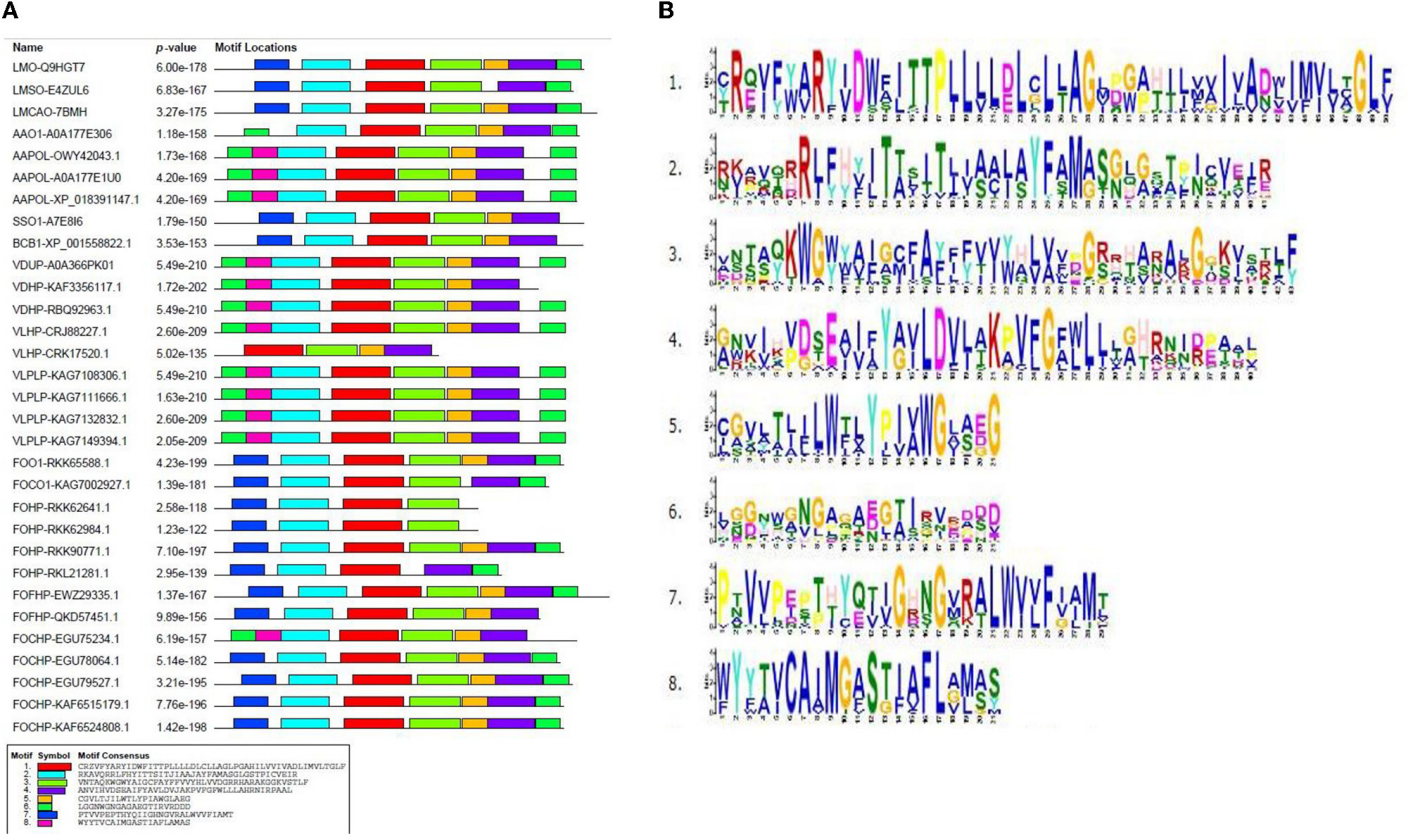
Conserved motifs in opsin protein using MEME program; **(A)** identified conserved motifs in opsin protein across all isoforms with their *P*-value, **(B)** sequence logo of these motifs.

The conserved motifs present in fungal species were determined by subjecting the amino acid sequence of the opsin protein to the MEME program. This analysis represented the fluctuation of motifs in the opsin protein during evolution. One highly conserved motif (motif 1) was found in all species. Further investigation also revealed that the conserved domain BACTERIAL_OPSIN_1 was located within motif 1. The second conserved domain, BACTERIAL_OPSIN_RET, which was found in two isoforms of *L. maculans*, three isoforms of *A. alternata, S. sclerotiorum*, and *B. cinerea*, was located within motif 4, whereas this domain of two isoforms of *F. oxysporum* was not detected within these motifs (Figure 4).

### 2.6. Consensus sequences of conserved motifs

According to the conserved motifs and conserved domains analyses, two motifs (1 and 4) were shared in almost all isoforms, which illustrated the importance of these motifs in the opsin protein across all fungal species. Then, Weblogo 3 was used to construct the consensus sequences of these two motifs. Motif 1 was 50 aa long and was present across all species. Amino acid residue number 12 (tryptophan) was conserved in all species except for four species of *F. oxysporum* (FOHPF-QKD57451.1, FOHPF-EWZ29335.1, FOHP-RKK62641.1, and FOHP-RKK62984.1). In contrast, amino acid residue number 24 (cysteine) was more conserved in these four isoforms of *F. oxysporum* as well as two other isoforms of *F. oxysporum* (FOHP-RKL21281.1, FOHPC-EGU78064.1) and two species of *Verticillium* (*V. dahliae* and *V. longisporum*) (Supplementary Figure S4).

Motif 4 was 40 aa long and was present in all isoforms, except for the two isoforms of *F. oxysporum* (FOHP-RKK62641.1 and FOHP-RKK62984.1) (Supplementary Figure S5). The amino acid residue number 13 (tyrosine) was entirely conserved in all isoforms.

### 2.7. Features of the secondary and tertiary structures and gene structure

The prediction of the secondary structure features was performed using Jpred4 software and Proteus2 software. According to this prediction, the lowest and highest percentages of helix content were detected in FOHP-RKL21281.1 (35 %) and FOHPF-QKD57451.1 (74 %), respectively. The percentage of helix content of other isoforms ranged from 43 % to 64 %. Most varieties of beta-sheet content and coil content were observed in *F. oxysporum*: isoform FOHP-RKK62641.1 did not possess any beta-sheets, whereas the highest percentage of beta-sheet belonged to isoform FOHP-RKL21281.1 (18 %); the highest content of coil also belonged to isoform FOHP-RKL21281.1 (48 %), and isoform FOHPF-QKD57451.1 had the lowest content of coil (25 %) (Table 3).

**Table 3:** The prediction of the secondary structure (helix content, beta-sheet content, coil content) (using Jpred4) and tertiary structure (ligands and pockets) (using SWISS-MODEL and P2Rank) of opsin protein of all isoforms.

**Name**	**Helix content %**	**Beta sheet content %**	**Coil content %**	**Number of ligands**	**Number of pockets**
LMO-Q9HGT7	55	6	39	11+ 2OLA	4
LMSO-E4ZUL6	54	8	38	10 LFA + 2 OLA	4
LMCAO-7BMH	56	5	40	-	-
AAO1-A0A177E306	54	10	36	5	6
AAPOL-OWY42043.1	51	11	38	2 (LFA7,14)	-
AAPOL-A0A177E1U0	49	10	41	2 (LFA7,14)	6
AAPOL-XP_018391147.1	50	10	40	2 (LFA7,14)	6
SSO1-A7E8I6	47	6	47	1 (LFA6)	4
BCB1-XP_001558822.1	56	11	34	1 (LFA6)	6
VDUP-A0A366PK01	51	10	39	1 (LFA7)	5
VDHP-KAF3356117.1	64	7	29	-	-
VDHP-RBQ92963.1	50	11	39	1 (LFA7)	5
VLHP-CRJ88227.1	51	10	38	1 (LFA7)	5
VLHP-CRK17520.1	59	15	26	1 (LFA7)	5
VLPLP-KAG7108306.1	51	10	39	1 (LFA7)	5
VLPLP-KAG7111666.1	51	11	39	1 (LFA7)	5
VLPLP-KAG7132832.1	51	10	39	1 (LFA7)	5
VLPLP-KAG7149394.1	51	10	38	1 (LFA7)	5
FOO1-RKK65588.1	55	11	33	2	10
FOCO1-KAG7002927.1	57	11	32	3 (LFA1,4,21)	10
FOHP-RKK62641.1	64	0	36	0	7
FOHP-RKK62984.1	60	4	36	0	9
FOHP-RKK90771.1	55	12	33	2 (LFA4, 21)	10
FOHP-RKL21281.1	35	18	48	0	9
FOHPF-EWZ29335.1	43	10	46	0	7
FOHPF-QKD57451.1	74	2	25	0	7
FOHPC-EGU75234.1	45	16	39	1 (LFA7)	4
FOHPC-EGU78064.1	44	12	44	0	8
FOHPC-EGU79527.1	52	10	38	2 (LFA4,21)	10
FOHPC-KAF6515179.1	56	7	36	2 (LFA4,21)	10
FOHPC-KAF6524808.1	55	11	34	2 (LFA4,21)	10

The tertiary structure of all isoforms was predicted using SWISS-MODEL software. The conformational changes were observed in four isoforms of *F. oxysporum* (FOHPF-QKD57451.1, FOHPF-EWZ29335.1, FOHP-RKK62641.1, and FOHP-RKK62984.1) (Supplementary Figure S3).

The analysis of exon-intron structure led to insight into the evolution of this gene. Introns were not identified across all isoforms.

### 2.8. The topography of the PPI networks, GO, and binding site analysis of the opsin protein

**Figure 5:**
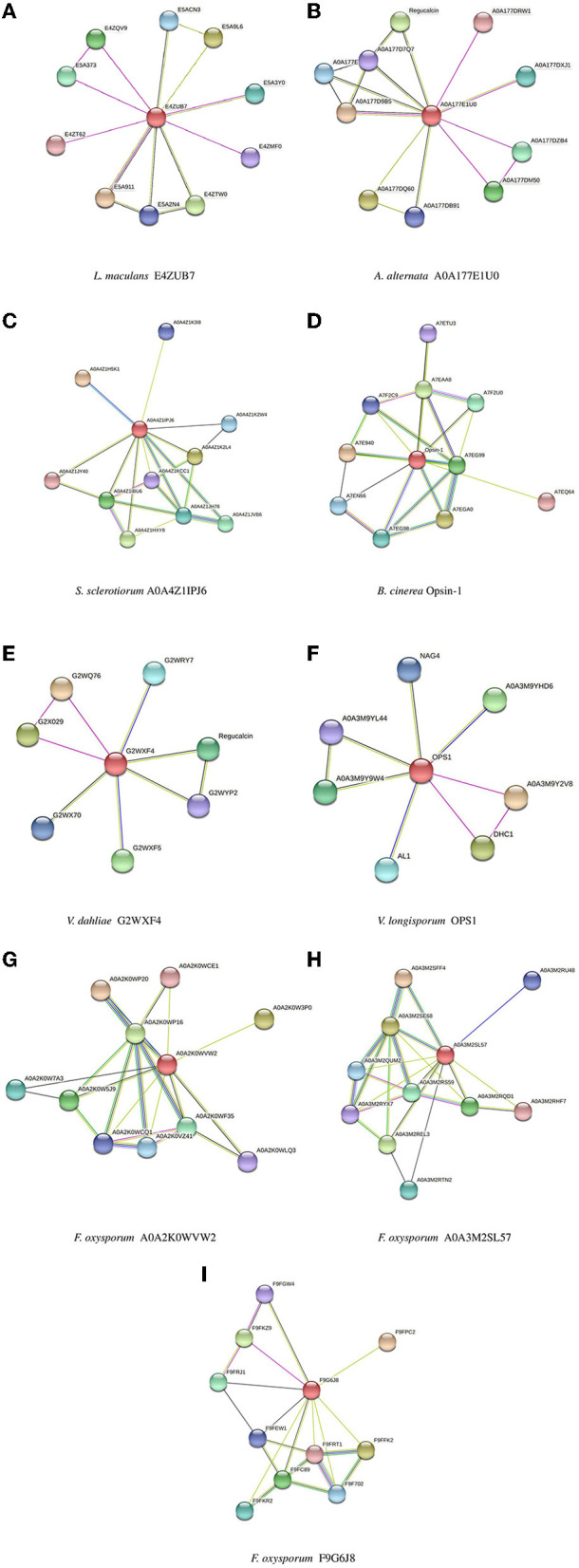
Protein-Protein interaction networks established by STRING for all isoforms. **(A)** *L. maculans*, **(B)** *A. alternata*, **(C)** *S. sclerotiorum*, **(D)** *B. cinerea*, **(E)** *V. dahliae*, **(F)** *V. longisporum*, **(G, H)** *F. oxysporum*, **(I)** *F. oxysporum* (FOHPC-EGU75234.1).

Biomolecular networks provide information regarding the molecular functions and interpretation of genomic variation. There are several biomolecular networks according to different purposes and scopes, including (1) networks of gene-gene regulatory events in transcription, networks of kinases/phosphatases, networks of metabolites and (2) networks of protein-protein interactions. Protein–protein interactions can occur under various physiological conditions and in different cell types. Proteins can also interact with each other indirectly, such as through a transcription factor that regulates gene expression and the production of another protein (Szklarczyk et al., 2019, 2021). The network of protein–protein interactions of the opsin protein of all isoforms was constructed using STRING software (version 11.5) (Figure 5).

Gene ontology (GO) is introduced as a common language in many organisms. GO is used to investigate and describe the function of proteins. The combination of gene ontology assessment and protein–protein interaction analysis enables the prediction of functions for unknown proteins. The three categories of GO are as follows: (1) biological process, which demonstrates the contribution of genes or proteins to specific biological events; (2) molecular function, which highlights the biochemical activities of the protein, such as ligand binding ligands, and (3) cellular component, which identifies the active location of a protein within cells. There is still a lack of knowledge regarding each category of GO for several proteins (Ashburner et al., 2000; Deng et al., 2004; Jain and Bader, 2010). An investigation into GO was conducted across all isoforms using STRING software (version 11.5) (Supplementary Table S1). Gene set enrichment analysis using STRING software showed no significant results.

The binding sites of the opsin protein were investigated using the P2Rank program. The total number of ligands in *L. maculans* ranged between 12 and 13, along with four pockets. Two ligands and six pockets were observed in three isoforms of *A. alternata*, and isoform AAO1-A0A177E306 of *A. alternata* possessed five ligands and six pockets. One identical ligand was observed in two species, *S. sclerotiorum*, and *B. cinerea*; they showed four and six pockets, respectively. *V. dahliae* and *V. longisporum* also possess a similar ligand with five pockets. The number of ligands and pockets in *F. oxysporum* ranged between 0 and 3 and 4 and 10, respectively (Table 3).

## 3. Discussion

### 3.1. Opsin characterization

The initial step in fungal-plant interactions is the recognition of the appropriate plant host. Various signals (chemical or physical signals) are perceived by fungi and in turn, they respond by differentiation, movement to an appropriate infection site, and formation of invasion-related structures (Kumamoto, 2008; Bonfante and Genre, 2010). For example, *Magnaporthe grisea*, the causative agent of rice blast, responds to contact with an appropriate surface by producing structures named appressoria. GPCR which is encoded by *PTH11* in this fungus, interacts with G proteins. Three subunits of G proteins (3 Gα subunits, 2 Gβ subunits, and one Gγ subunit) are encoded by the genome of *M. grisea*. And it has been reported that the formation of appressoria will be incomplete under the condition of deletion of each of these subunits (Nishimura et al., 2003; Kulkarni et al., 2005). In this research, opsin, a member of the rhodopsin family (the first family of GPCRs), of six phytopathogenic ascomycetous fungi (*L. maculans, A. alternata, S. sclerotiorum, B. cinerea, V. dahliae, V. longisporum*, and *F. oxysporum*) was studied.

Protein domains are evolutionarily conserved sequences with the basic components of protein structure and function (Murzin et al., 1995; Buljan et al., 2010). Evolutionary processes can lead to the emergence of new domains within proteins. Consequently, these new domains may contribute to the development of novel functions in organisms, potentially expanding their functional repertoire (Itoh et al., 2007). In comparison, fungi possess different numbers of domains than other organisms. Domain duplication during recombination causes the expansion of the domain in the fungal genome (Björklund et al., 2006). A study identified the bacteriorhodopsin domain in the microbial opsin homolog Sop1 in *S. sclerotiorum* (Lyu et al., 2016). Seven putative transmembrane helix domains have been reported in an opsin gene, *nop-1*, in *N. crassa* (Bieszke et al., 1999a). BACTERIAL_OPSIN_1 and BACTERIAL_OPSIN_RET are the two identified domains in this study. Most isoforms of studied fungi with the same domain/s were grouped in the same clade in the phylogenetic tree, which is hypothesized to have an identical biological function. Further analysis revealed that BACTERIAL_OPSIN_1 was located within the conserved motif of all species, indicating the importance of this conserved domain during evolutionary events. Bacterial opsins play a role in Light-dependent ion transport and sensory in a family of halophilic bacteria. These proteins are integral membrane proteins that contain seven transmembrane domains. The two classes of these proteins are as follows: (1) light-driven proton pumps, bacteriorhodopsin and archaerhodopsin and (2) a light-driven chloride pump, halorhodopsin (Oesterhelt, 1989; Soppa et al., 1993; Purschwitz et al., 2006).

The membrane composition influences the membrane protein's function and stability (Lee, 2004; Hunte and Richers, 2008; Phillips et al., 2009). Tryptophan is an amino acid that highly affects protein folding (Sanchez et al., 2008; Fiedler et al., 2010). Tryptophan is also involved in anchoring the hydrophobic transmembrane part across the membrane and causing hydrophobic mismatch, which highly affects the change of the transmembrane segment and the membrane structure (Sun et al., 2008). Transmembrane proteins also possess tryptophan amino acid near the end of the transmembrane helices. The above-mentioned functions of tryptophan are also reported in transmembrane proteins. Therefore, it is presumed that this amino acid could influence the function of opsin proteins as one of the transmembrane proteins (Von Heijne, 2007; De Jesus and Allen, 2013). Our study revealed that tryptophan was highly conserved across all isoforms (Supplementary Figure S2). Consensus sequence analysis of motif 1 demonstrated that tryptophan amino acid (residue 12) was highly conserved among all clades of the phylogenetic tree except for clade 2 (Supplementary Figure S4). According to the secondary and tertiary structure analysis of isoforms in clade 2 (FOHPF-QKD57451.1, FOHPF-EWZ29335.1, FOHP-RKK62641.1, and FOHP-RKK62984.1), the conformation of these isoforms has changed, confirming the importance of tryptophan regarding its effect on the structure of the protein membrane. Hence, it is hypothesized that these isoforms also have different functions.

#### 3.1.1. Subcellular localization

Proteins are transported to the appropriate location to initiate their functions. Therefore, understanding protein localization provides important information concerning the function of the protein and its interaction with other proteins (Sadowski et al., 2008; Cosson et al., 2013; Peng and Gao, 2014). Subcellular localization prediction demonstrated that all isoforms were located in the endoplasmic reticulum membrane, except for seven isoforms of *Verticillium*, which were located in the mitochondrial membrane. The endoplasmic reticulum is a part of the secretory pathway while mitochondrion depends on another pathway (routes of lipid distribution). Therefore, it could be suggested that isoforms that possess the same subcellular localization are involved in the same cellular pathway (Jan et al., 2014; Acoba et al., 2020; Tamura et al., 2020).

The GPCRs are seven-transmembrane proteins. To initiate the intracellular protein transport of GPCRs, their insertion into the endoplasmic reticulum is required. For this purpose, two various pathways are used in membrane proteins with an extracellular N-terminal tail, such as GPCRs; the first group uses the uncleaved signal anchor sequence, which is the first transmembrane domain of the receptor. Moreover, the second subset possesses cleavable signal peptides. According to the statistical analysis, the nature of the N-terminus of the GPCRs may correlate with the presence of the signal peptide. Possessing the signal peptides is required for the GPCRs, for which the post-translational translocation of their N-terminus is either impaired or impossible. Signal peptide prediction in this study shows that only one isoform (*B. cinerea*) possesses the signal peptide in its N-terminus, which may impact the function of this isoform. Moreover, the transmembrane topology of these isoforms displayed N-terminus tail differences among these isoforms, such as VLHP-CRK17520.1. Various N-terminus tails and numbers of the transmembrane have been found among the isoforms of *F. oxysporum*, and also the conformational change within four isoforms of this species (FOHPF-QKD57451.1, FOHPF-EWZ29335.1, FOHP-RKK62641.1, and FOHP-RKK62984.1) has been found. It has been hypothesized that these varieties of the N-terminus, the number of transmembranes, and the conformational change might affect the function of these isoforms (Supplementary Figure S3).

#### 3.1.2. Phylogenetic tree

Two of the most important pieces of information can be provided by reconstructing a phylogenetic tree: first, the evolutionary history of living organisms on earth; and second, the evidence history of the climate and geological development of earth (Uncu et al., 2015). The evolutionary relationship among these fungi was investigated by creating a phylogenetic tree.

##### 3.1.2.1. Maximum likelihood tree

ClustalW and bootstrap values of 1,000 iterations were used. Six major groups and six subgroups in the phylogenetic tree indicated divergent evolution among these isoforms. The phylogenetic tree also showed that most isoforms with identical domains were grouped in the same clade. However, proteins with the same domain can function differently due to mutations in their sequences (Cannon et al., 2004). This method helps determine the tree topology and branch lengths with the greatest likelihood. The likelihood is calculated for several models of nucleotide or amino acid substitution processes for each residue in an alignment (Felsenstein, 1981).

Most isoforms of *F. oxysporum* were grouped in the first and second clades; *S. sclerotiorum* and *B. cinerea* were grouped in the third clade; the fourth clade included *L. maculans* and one isoform of *A. alternata* (AAO1-A0A177E306); three other isoforms of *A. alternata* and one isoform of *F. oxysporum* (FOHPC-EGU75234.1) belonged to the fifth clade; the last clade included two species of *Verticillium* (*V. dahliae* and *V. longisporum*).

##### 3.1.2.2. Maximum parsimony tree

A phylogenetic tree with the maximum parsimony method was created to precisely analyze these isoforms. The above-mentioned alignment file was also used for creating this phylogenetic tree. In comparison with the first phylogenetic tree, main clades were also observed in the second phylogenetic tree. Each clade was divided into several sub-groups in the second phylogenetic tree. It could be presumed that isoforms in these subgroups were evolutionary close to each other (Supplementary Figure S6).

#### 3.1.3. Post-translational modifications

One of the post-translational modifications of proteins in the cell is glycosylation. Asparagine (N)-linked glycosylation of proteins is formed by the covalent binding of oligosaccharides onto asparagine residues of polypeptide chains (Koch, 1992; Landolt-Marticorena and Reithmeier, 1994; Kukuruzinska and Lennon, 1998; Shental-Bechor and Levy, 2008; Aebi et al., 2010; Schwarz and Aebi, 2011; Aebi, 2013). Translational N-glycosylation of membrane proteins occurs in the endoplasmic membrane and Golgi, where the processes of folding and maturation of membrane proteins take place and influences protein quality control (Chandler and Costello, 2016). Although glycans influence the above-mentioned functions, the role of glycans in these functions, such as folding proteins, is ambiguous. It has been reported that glycans function as chaperones and impact protein folding (Mitra et al., 2006; Shental-Bechor and Levy, 2008). The glycosylation assessment in this study illustrated that the isoforms with N- glycan sites were grouped in the same clade in the phylogenetic tree, suggesting that these isoforms might be involved in the same functions. There are several exceptions regarding N-glycosylation in the phylogenetic tree. *B. cinerea* possesses a peptide signal instead of N-glycosylation that might act differently. Two isoforms of *Verticillium* (VDHP-KAF3356117.1 and VLHP-CRK17520.1) do not possess any N-glycan sites while they are grouped with other isoforms of this fungus. Isoform FOHPC-EGU75234.1 is a subgroup of *A. alternata* and possesses two N-glycan sites. The location of the glycans affects the stabilization of proteins (Shental-Bechor and Levy, 2008). There is a hypothesis that these differences in N-glycosylation might influence the function, stabilization, and other characteristics of these fungi.

#### 3.1.4. Protein structures

The primary structure, the linear amino acid sequence, and the description of the amino acid sequence in the polypeptide chain are the four main stages of protein structure characterization. The side chains of these amino acids can be polar, non-polar, or positive or negative charged, for example. These variations are crucial to the structure of proteins because they help retain a length of protein in a certain shape or conformation and connect side chains together. A polypeptide chain's secondary structure is its local spatial shape. The protein chain's adjacent amino groups and carboxyl groups form hydrogen bonds to form this structure. It is known as alpha helices and beta sheets. Tertiary structure is the three-dimensional arrangement of monomeric and multimeric protein molecules. It is also called a polypeptide. A quaternary structure is the three-dimensional structure of a macromolecule protein (Sun et al., 2004; Hartl and Hayer-Hartl, 2009). The secondary structure is made up of β-sheets and α-helices. The tertiary structure of a protein is created through interactions between the side chains of the amino acids. The tertiary structure of a protein is influenced by the polarity and charge of amino acid constituents. Every modification to a protein's fundamental structure and every instance of protein misfolding have an impact on the protein's structure. The functions of proteins are impacted by their structure. (Sun et al., 2004; Moghadam et al., 2016). The secondary and tertiary structures of all isoforms were predicted in this study. The percentage of α-helix content varies between 35% and 74 % among all isoforms, and the β-sheet content ranges between 0 and 18 % across all isoforms. A conformational change of the tertiary structure was observed in four isoforms of *F. oxysporum* (FOHPF-QKD57451.1, FOHPF-EWZ29335.1, FOHP-RKK62641.1, and FOHP-RKK62984.1).

Fungi have short introns and long exons compared to their mammalian counterparts (Kupfer et al., 2004). Different selections and mutations on introns affect the intron densities. Losing the amount of intron over a specific time could reflect general evolution (Lynch, 2002; Denoeud et al., 2010; Venkatesh et al., 2014). Gene structure analysis in this study did not show any introns in any of the studied isoforms. It could be due to an evolutionary event. A lower number of introns in a gene is interpreted as genetic evolution (Jacob and Smith, 2017; Naro and Sette, 2017).

#### 3.1.5. Protein-protein networks

The most crucial information regarding the biological and cellular functions of proteins is provided by the prediction of protein-protein interactions. In a protein–protein interaction network, two or more proteins are bound together to accomplish their functions. It means that the function and activity of proteins are often modulated by other connected proteins in the network of their protein-protein interactions (Phizicky and Fields, 1995). Protein E4ZUB7 from *L. maculans* and protein A0A177E1U0 from *A. alternata* showed an identical connection with RRM domain-containing protein. These proteins of *L. maculans* and *A. alternata*, along with proteins G2WXF4 and OPS1 from *V. dahliae* and *V. longisporum*, respectively, displayed similar connections with Dynein heavy chain, cytoplasmic. Protein E4ZUB7 from *L. maculans* and OPS1 from *V. longisporum* illustrated another identical connection with SGL domain-containing protein. Protein A0A177E1U0 from *A. alternata* and protein G2WXF4 from *V. dahliae* demonstrated a similar connection with Regucalcin. Proteins of two species of *Verticillium* (*V. dahliae* and *V. longisporum*) exhibit a similar connection with SBDS domain-containing protein in the network of their protein–protein interactions. Protein A0A4Z1IPJ6 from *S. sclerotiorum* and protein Opsin-1 from *B. cinerea*, and these two proteins (A0A2K0WVW2, A0A3M2SL57) from different isoforms of *F. oxysporum* exhibited two identical connections with two proteins, Amino_oxidase domain-containing protein and PAS domain-containing protein. These proteins, A0A4Z1IPJ6 from *S. sclerotiorum* and protein Opsin-1 from *B. cinerea*, and A0A2K0WVW2 from *F. oxysporum*, also demonstrated a connection with Photolyase/cryptochrome alpha/beta domain-containing protein. The protein of *S. sclerotiorum* (A0A4Z1IPJ6) illustrated one more similar connection, the same as two proteins (A0A2K0WVW2, A0A3M2SL57) from different isoforms of *F. oxysporum* with Bifunctional lycopene cyclase/phytoene synthase. These two proteins of *F. oxysporum* had the same connection with Cryptochrome DASH. GO analysis of all isoforms illustrates that there is still a challenge in several studied isoforms whose functions are unknown (Supplementary Table S1). The gene set enrichment analysis did not show any significant results among all isoforms.

Endogenous peptides or protein ligands activate most GPCRs in humans. The different structures of the ligand-binding pocket between GPCRs and rhodopsin have illustrated that rhodopsins are not only bound to the ligands but also held in the pocket, which is required for the activation of rhodopsins by the photon-triggered isomerization of retinal, while the other GPCRs can be activated by binding to the ligands. The modes of peptide ligands vary in the receptors even within the same phylogenetic family, which indicates the specificity of their physiological function (Zhou et al., 2012; Kaiser and Coin, 2020). Our study reveal that most isoforms that are grouped in the same clade in the phylogenetic tree possess the same ligand and pocket.

### 3.2. Fungi characterization

*L. maculans* belongs to the Dothideomycetes class and the Pleosporales order. Several other important pathogens are in this order, such as those from the genus *Alternaria*. Pseudothecia production has been observed among most fungi of the Dothideomycetes class, forming either a single or a group of fruiting bodies. Fungi in the Dothideomycetes class have varieties of host plants, but their life cycles and pathogenicity strategies are often similar (Eyal, 1999; Nolan et al., 1999; Kaczmarek and Jedryczka, 2012). *L. maculans* is introduced as a hemibiotrophic fungus. Several plants were suggested as hosts for *L. maculans* in the past, such as *Phaseolus* sp., *Humulus* sp., *Swertia perennis, Teucrium* sp., and *Artemisia campestris*. Afterward, reports highlighted crucifers and mainly *Brassica* crops as the hosts of *L. maculans* (Mendes-Pereira et al., 2003). Fungi belonging to the genus *Leptosphaeria* can cause allergic responses of types I and II in humans (Hasnain, 1993). The genus *Alternaria* from the Dothideomycetes class mainly includes saprophytic fungi; some species are plant pathogens. There is a broad host range, such as cereals, ornamentals, oil crops, vegetables (cauliflower, broccoli, carrot, potato, tomato), citrus, and apple. Several species of *Alternaria* cause cancer in mammals. Moreover, spores of this genus are also introduced as airborne allergens (Meena et al., 2010). According to the similarity among fungi of the Dothideomycetes class, it has been hypothesized that isoforms AAO1-A0A177E306 and isoforms of *L. maculans*, which are grouped in one clade in the phylogenetic tree, can play a similar role in sporulation and have the identical growth pathway. They could also be considered allergenic pathogens for humans. Although the other isoforms of *A. alternata* may have different sporulation pathways, they may also have a less allergic effect on humans.

Two necrotrophs and soilborne fungi, *S. sclerotiorum* (white mold fungus) and *B. cinerea* (gray mold fungus) are very close taxonomically. Their host range is broader than that of most other plant pathogens (Bolton et al., 2006; Williamson et al., 2007). Both fungi produce sclerotia that survive in the soil. They also produce melanized sclerotium, which is involved in their lifecycle by germinating vegetatively or sexually. *Botrytis* spp. also belongs to the Sclerotiniaceae family. Proteins encoded by the genomes of *B. cinerea* and *S. sclerotiorum* are 83% identical, which points to a very close relationship between these two genera (Groves and Loveland, 1953; Nees, 1973; Amselem et al., 2011). Despite many similarities in developmental and physiological features between these two fungi, they vary in their regulation and potential for sporulation. *S. sclerotiorum* produces ascospores and is dispersed via airborne spores, whereas *B. cinerea* produces ascospores, but its dispersal is predominantly via conidia. Furthermore, they are different in the regulation of sexual sporulation, with *S. sclerotiorum* being homothallic while *B. cinerea* is heterothallic (Bolton et al., 2006; Williamson et al., 2007). Most similarities between these two fungi confirm the phylogenetic analysis of this study, and the above-mentioned differences that have been found in this study suggest that the opsin protein may be involved in the melanized sclerotium pathway and can have an identical function in their growth and sporulation.

The widespread distribution of *Fusarium* species has been reported on plants, in soil, and in water as parasites, endophytes, or saprophytes. *Fusarium* causes several varieties of diseases in on-field, horticultural, ornamental, and forest crops, such as wilts, blights, rots, and cankers. Varieties of toxic secondary metabolites are produced by fusaria, such as trichothecenes and fumonisins, that are involved in the contamination of agricultural products; trichothecenes can also be a virulence factor in plant diseases. A few *Fusarium* species have been introduced as opportunistic human pathogens that cause corneal infections (O'Donnell et al., 2004). Life cycle, specialization, host adaptation, and specificity differ among *Fusarium* species. *F. oxysporum* has a broad host range, including monocotyledonous and dicotyledonous plants. Moreover, it can also infect immunocompromised patients and other mammals (O'Donnell et al., 2004; Ortoneda et al., 2004). *F. oxysporum* is an anamorphic species. It is a heterogeneous and polytypic morphospecies that is introduced as the most abundant soilborne fungus (Waalwijk et al., 1996; O'Donnell and Cigelnik, 1997; Snyder and Hansen, 2013). Most of the *F. oxysporum* isoforms were grouped in the same clade in the phylogenetic analysis, which displays similarity within this forma. Despite these similarities, several subgroups have been observed in this phylogenetic tree analysis of the opsin protein, which points to the different performances of the opsin protein within this species, demonstrating different recognition and function strategies of this protein on different hosts. It has been hypothesized that one isoform of *F. oxysporum* (FOHPC-EGU75234.1), which was grouped with three isoforms of *A. alternata*, can not be an allergenic pathogen to humans.

The genus *Verticillium* involves soilborne plant pathogenic species that cause vascular wilt diseases on a wide range of plants, especially in the temperate and subtropical regions of the world (Fradin and Thomma, 2006). The *Verticillium* genus comprises two plant pathogenic species: *V. dahliae*, which forms microsclerotia. *V. albo-atrum*, which produces dark resting mycelium, and the other four species are saprophytes and non-pathogenic species (*V. nigrescens, V. nubilum, V. tricorpus*, and *V. theobromae*) (Barbara and Clewes, 2003). *V. dahliae* causes verticillium wilt in a wide range of host plants, including woody, dicotyledonous species of herbaceous annuals and perennials (Bhat and Subbarao, 1999). Subsequent studies have revealed the occurrence of parasexual hybridization between *V. albo-atrum* and *V. dahliae* to evolve *V. longisporum* (Karapapa et al., 1997; Zeise and Von Tiedemann, 2001). Several studies have illustrated the host specificity of *V. longisporum* in brassicaceous hosts, whereas *V. dahliae* is restricted to non-brassicaceous hosts (Zeise and Von Tiedemann, 2001, 2002a,b). The phylogenetic analysis of this study separated *Verticillium* species from other fungi, demonstrating higher similarity between these two species. These two species were separated within their clade in this phylogenetic analysis of the opsin protein, suggesting that this protein acts differently among these two species of the genus *Verticillium* concerning plant–pathogen interaction, pathogenicity, and sporulation.

## 4. Material and methods

### 4.1. Collection of opsin protein sequences

Protein sequences of opsins from *L. maculans, A. alternata, S. sclerotiorum, B. cinerea, V. dahliae, V. longisporum*, and *F. oxysporum* were retrieved from the NCBI and UniProt databases. Their gene IDs are listed in Supplementary Table S2. Then, BLASTP was conducted across the proteomes of the above-mentioned fungi using the opsin protein sequences. The identified opsin proteins were analyzed for the presence of conserved domains using the Pfam [a protein families database that identifies proteins by using two alignments and a profile hidden Markov model (HMM)] (Finn et al., 2014), SMART (a database that is used to identify and annotate the genetically mobile domains and analysis of their architectures) (Schultz et al., 2000), ENSEMBLE (a comprehensive database that provides information on the annotation of individual genomes of many species such as plants and microorganisms) (Birney et al., 2004), Conserved Domain Database (CDD) (the annotation of protein sequences is provided by this resource along with the location of conserved domain footprints) (Marchler-Bauer et al., 2011), and PROSITE (two signatures (patterns and generalized profiles) are used in this database to identify conserved regions) (Hulo et al., 2006) databases. Therefore, these databases provided comprehensively conserved domain analyses by applying different alignment and annotation methods. The position and scale of protein domains were drawn using Domain Graph (DOG) software (http://dog.biocuckoo.org/).

### 4.2. Physicochemical properties of opsin homologs

Physicochemical properties, including isoelectric point (pI), molecular weight (Mw), instability index (II), grand average of hydropathicity (GRAVY), and aliphatic index (AI), were investigated for identified opsin proteins using the ProtParam tool available on the ExPASy website (https://web.expasy.org/protparam/) and EMBOSS Pepstats (https://www.ebi.ac.uk/Tools/seqstats/emboss_pepstats/). The coevolution of amino acids was investigated using MISTIC (Mutual Information Server to Infer Coevolution) (http://mistic.leloir.org.ar/index.php).

### 4.3. Subcellular localization and transmembrane domains

Subcellular localization of opsin proteins was predicted using Loctree3 (https://rostlab.org/services/loctree3/). Nuclear localization signal prediction was conducted using NucPred (https://nucpred.bioinfo.se/cgi-bin/single.cgi). The integral transmembrane (TM) domains were investigated using PolyPhobius (https://phobius.sbc.su.se/poly.html) and Protter (http://wlab.ethz.ch/protter/start/). The prediction of signal peptide cleavage sites was performed using TargetP-2.0 (https://services.healthtech.dtu.dk/services/TargetP-2.0/).

### 4.4. Phylogenetic analysis

Multiple sequence alignment of the opsin sequences of these fungal species was performed using the ClustalW tool in MEGAX. Then, the alignment file was used to create a phylogenetic tree alongside with Maximum Likelihood method (1000 repeats) with the parameters of the Jones-Taylor-Thornton (JTT) matrix-based model, uniform rates among sites, and partial deletion of gaps in MEGAX software. Opsin sequences of one species of yeast (*Cryptococcus neoformans*) and one species of archaea (*Haloarcula sinaiiensis*) were downloaded from the UniProt database to be used as outgroup in the phylogenetic tree. The alignment file was also used to generate a phylogenetic tree, alongside with Maximum parsimony method in MEGAX. In the second phylogenetic tree the calculation of bootstrap values was also at 1000 iterations with the parameters of the Subtree-Pruning-Regrafting (SPR) method and partial deletion of gaps in MEGAX software. Two above-mentioned outgroups were also used in the second phylogenetic tree.

### 4.5. Identification of conserved motifs and sequence logo

Conserved motifs of opsin proteins across all fungal species were predicted using the MEME (https://meme-suite.org/meme/) tool with the maximum number of eight motifs, and the other parameters were set to default. The sequence logos of conserved motifs were generated using Weblogo 3 (http://weblogo.threeplusone.com/).

### 4.6. Protein and gene structures of opsin homologs

The features of the secondary structure were predicted using Jpred4 software (https://www.compbio.dundee.ac.uk/jpred/) and Proteus2 software (http://www.proteus2.ca/proteus2/), and the SWISS-MODEL software (https://swissmodel.expasy.org/) was used for the prediction of the fatures of the tertiary structure of opsin proteins in all fungal species. To investigate the exon and intron structures, blastx was performed at NCBI for each isoform.

### 4.7. Protein-protein interactions and gene ontology (GO) analyses

Protein–protein interactions (PPI) of the opsin protein in all fungal species were constructed using String software (https://string-db.org/). Afterward, constructed networks were investigated considering the GO annotation, including Biological Processes (BP), Molecular Functions (MF), and Cellular Component (CC). String software was also used for Gene set enrichment analysis across all isoforms. Binding sites, including ligand-binding pockets of the opsin protein, were predicted using P2Rank (https://prankweb.cz/).

## Data availability statement

The datasets presented in this study can be found in online repositories. The names of the repository/repositories and accession number(s) can be found in the article/Supplementary material.

## Author contributions

AM and MM: conception, design, and data curation of the study. AM: methodology, investigation, validation, software, supervision, review, and editing. MM: formal analysis, methodology, software, investigation, and writing. All authors contributed to the manuscript revision and read and approved the submitted version.

## Footnotes

^1^ https://www.statista.com/statistics/263930/worldwide-production-of-rapeseed-by-country/

## Supplementary material

Supplementary Figure 1 Comparison of the amino acid composition of opsin protein in all isoforms; **(A)** *L. maculans*, **(B)** *A. alternata*, **(C)** *S. sclerotiorum*, **(D)** *B. cinerea*, **(E)** *V. dahliae*, **(F)** *V. longisporum*, **(G–I)** *F. oxysporum*. The y-axis shows the percentage of amino acids of opsin protein in each isoform; the X-axis presents the name of all amino acids.

Supplementary Figure 2 The coevolutionary analysis of the amino acids of opsin protein across all isoforms using Mistic. Labels in the outer circle illustrate the amino acid codes of the opsin protein of all isoforms. Red boxes show the highly conserved positions and blue boxes indicate less conserved positions. Red arrows show highly conserved amino acid (Tryptophan).

Supplementary Figure 3 Transmembrane domains analysis of the opsin protein across all isoforms using Protter; **(A)** *L. maculans*, **(B)** *S. sclerotiorum*, **(C)** *B. cinerea*, **(D)** *A. alternata*, **(E)** *V. dahliae*, **(F)** *V. longisporum*, and **(G)** *F. oxysporum*. Red circles show N-glycan sites.

Supplementary Figure 4 Consensus sequences of motif 1 in opsin protein across all isoforms using Weblogo 3; **(A)** isoforms of *F. oxysporum* (FOHPC-EGU79527.1, FOHPC-KAF6515179.1, FOHP-RKK90771.1, FOO1-RKK65588.1, FOHPC-KAF6524808.1, FOCO1-KAG7002927.1); **(B)** isoforms of *F. oxysporum* (FOHP-RKL21281.1, FOHPC-EGU78064.1); **(C)** isoforms of *F. oxysporum* (FOHPF-QKD57451.1, FOHPF-EWZ29335.1); **(D)** isoforms of *F. oxysporum* (FOHP-RKK62641.1 and FOHP-RKK62984.1); **(E)** *S. sclerotiorum*, and *B. cinerea*; **(F)** *L. maculans* and one isoform of *A. alternata* (AAO1-A0A177E306); **(G)** *A. alternata* and one isoform of *F. oxysporum* (FOHPC-EGU75234.1); **(H)** *V. dahliae* and *V. longisporum*.

Supplementary Figure 5 Consensus sequences of motif 4 in opsin protein across all isoforms using Weblogo 3; **(A)** isoforms of *F. oxysporum* (FOHPC-EGU79527.1, FOHPC-KAF6515179.1, FOHP-RKK90771.1, FOO1-RKK65588.1, FOHPC-KAF6524808.1, FOCO1-KAG7002927.1); **(B)** isoforms of *F. oxysporum* (FOHP-RKL21281.1, FOHPC-EGU78064.1); **(C)** isoforms of *F. oxysporum* (FOHPF-QKD57451.1, FOHPF-EWZ29335.1); **(D)** *S. sclerotiorum*, and *B. cinerea*; **(E)** *L. maculans* and one isoform of *A. alternata* (AAO1-A0A177E306); **(F)** *A. alternata* and one isoform of *F. oxysporum* (FOHPC-EGU75234.1); **(G)** *V. dahliae* and *V. longisporum*.

Supplementary Figure 6 Phylogenetic tree using MEGAX (Maximum Parsimony method). The maximum parsimony method presents a close evolutionary relationship of opsin protein across all isoforms.
