# Supplementary material for: Genome-wide identification of the opsin protein in Leptosphaeria maculans and comparison with other fungi (pathogens of Brassica napus)
Source: Front Microbiol. 2023 Aug 25;14:1193892. doi: 10.3389/fmicb.2023.1193892 (PMC10485269; doi:10.3389/fmicb.2023.1193892)
Supplement: Supplementary file 1 [file Table_1.docx]

**Supplementary Table 1.** GO assessment of all isoforms; biological processes and molecular functions.

| Name | Biological process | Molecular function |
| --- | --- | --- |
| LMO-Q9HGT7 | - | - |
| LMSO-E4ZUL6 | - | - |
| LMCAO-7BMH | - | - |
| AAO1-A0A177E306 | - | - |
| AAPOL-OWY42043.1 | - | - |
| AAPOL-A0A177E1U0 | - | - |
| AAPOL-XP_018391147.1 | - | - |
| SSO1-A7E8I6 | Carotenoid biosynthetic process, Protein-chromophore linkage, Response to light stimulus | Photoreceptor activity |
| BCB1-XP_001558822.1 | Carotenoid biosynthetic process, Protein-chromophore linkage, Response to light stimulus, Response to stimulus | Photoreceptor activity, Signaling receptor activity |
| VDUP-A0A366PK01 | - | - |
| VDHP-KAF3356117.1 | - | - |
| VDHP-RBQ92963.1 | - | - |
| VLHP-CRJ88227.1 | - | - |
| VLHP-CRK17520.1 | - | - |
| VLPLP-KAG7108306.1 | - | - |
| VLPLP-KAG7111666.1 | - | - |
| VLPLP-KAG7132832.1 | - | - |
| VLPLP-KAG7149394.1 | - | - |
| FOO1-RKK65588.1 | Protein-chromophore linkage, Carotenoid biosynthetic process, Response to light stimulus, Cellular protein modification process, Cellular response to stimulus | DNA photolyase activity, Photoreceptor activity |
| FOCO1-KAG7002927.1 | Protein-chromophore linkage, Carotenoid biosynthetic process, Response to light stimulus, Cellular protein modification process, Cellular response to stimulus | DNA photolyase activity, Photoreceptor activity |
| FOHP-RKK62641.1 | Protein-chromophore linkage, Carotenoid biosynthetic process, Response to light stimulus, Cellular protein modification process | DNA photolyase activity, Photoreceptor activity |
| FOHP-RKK62984.1 | Protein-chromophore linkage, Carotenoid biosynthetic process, Response to light stimulus, Cellular protein modification process | DNA photolyase activity, Photoreceptor activity |
| FOHP-RKK90771.1 | Protein-chromophore linkage, Carotenoid biosynthetic process, Response to light stimulus, Cellular protein modification process, Cellular response to stimulus | DNA photolyase activity, Photoreceptor activity |
| FOHP-RKL21281.1 | Protein-chromophore linkage, Carotenoid biosynthetic process, Response to light stimulus, Cellular protein modification process, Cellular response to stimulus | DNA photolyase activity, Photoreceptor activity |
| FOHPF-EWZ29335.1 | Protein-chromophore linkage, Carotenoid biosynthetic process, Response to light stimulus, Cellular protein modification process | DNA photolyase activity, Photoreceptor activity |
| FOHPF-QKD57451.1 | Protein-chromophore linkage, Carotenoid biosynthetic process, Response to light stimulus, Cellular protein modification process | DNA photolyase activity, Photoreceptor activity |
| FOHPC-EGU75234.1 | Protein unfolding, Protein folding, Response to oxidative stress | - |
| FOHPC-EGU78064.1 | Protein-chromophore linkage, Carotenoid biosynthetic process, Response to light stimulus, Cellular protein modification process, Cellular response to stimulus | DNA photolyase activity, Photoreceptor activity |
| FOHPC-EGU79527.1 | Protein-chromophore linkage, Carotenoid biosynthetic process, Response to light stimulus, Cellular protein modification process, Cellular response to stimulus | DNA photolyase activity, Photoreceptor activity |
| FOHPC-KAF6515179.1 | Protein-chromophore linkage, Carotenoid biosynthetic process, Response to light stimulus, Cellular protein modification process, Cellular response to stimulus | DNA photolyase activity, Photoreceptor activity |
| FOHPC-KAF6524808.1 | Protein-chromophore linkage, Carotenoid biosynthetic process, Response to light stimulus, Cellular protein modification process, Cellular response to stimulus | DNA photolyase activity, Photoreceptor activity |
