# Supplementary material for: Genome-wide identification of the opsin protein in Leptosphaeria maculans and comparison with other fungi (pathogens of Brassica napus)
Source: Front Microbiol. 2023 Aug 25;14:1193892. doi: 10.3389/fmicb.2023.1193892 (PMC10485269; doi:10.3389/fmicb.2023.1193892)
Supplement: Supplementary file 2 [file Table_2.docx]

**Supplementary Table 2.** Protein ID of isoforms from UniProt and their names in this study.

| Gene ID | Name |
| --- | --- |
| Q9HGT7_LEPMC Opsin OS=Leptosphaeria maculans | LMO-Q9HGT7 |
| E4ZUL6_LEPMJ Similar to opsin-1 OS=Leptosphaeria maculans | LMSO-E4ZUL6 |
| 7BMH_A Chain A, Opsin [Leptosphaeria maculans] | LMCAO-7BMH |
| A0A177E306_ALTAL Opsin-1 OS=Alternaria alternata | AAO1-A0A177E306 |
| OWY42043.1 putative opsin-like protein [Alternaria alternata] | AAPOL-OWY42043.1 |
| A0A177E1U0_ALTAL Putative opsin-like protein OS=Alternaria alternata | AAPOL-A0A177E1U0 |
| XP_018391147.1 putative opsin-like protein [Alternaria alternata] | AAPOL-XP_018391147.1 |
| A7E8I6_SCLS1 Opsin-1 OS=Sclerotinia sclerotiorum | SSO1-A7E8I6 |
| XP_001558822.1 Bop1 [Botrytis cinerea B05.10] | BCB1-XP_001558822.1 |
| A0A366PK01_VERDA Uncharacterized protein OS=Verticillium dahliae | VDUP-A0A366PK01 |
| KAF3356117.1 hypothetical protein VdG1_00232 [Verticillium dahliae VDG1] | VDHP-KAF3356117.1 |
| RBQ92963.1 hypothetical protein VDGD_02933 [Verticillium dahliae] | VDHP-RBQ92963.1 |
| CRJ88227.1 hypothetical protein BN1723_001539, partial [Verticillium longisporum] | VLHP-CRJ88227.1 |
| CRK17520.1 hypothetical protein BN1708_017586, partial [Verticillium longisporum] | VLHP-CRK17520.1 |
| KAG7108306.1 Protein like protein [Verticillium longisporum] | VLPLP-KAG7108306.1 |
| KAG7111666.1 Protein like protein [Verticillium longisporum] | VLPLP-KAG7111666.1 |
| KAG7132832.1 Protein like protein [Verticillium longisporum] | VLPLP-KAG7132832.1 |
| KAG7149394.1 Protein like protein [Verticillium longisporum] | VLPLP-KAG7149394.1 |
| RKK65588.1 Opsin-1 [Fusarium oxysporum] | FOO1-RKK65588.1 |
| KAG7002927.1 Opsin-1 [Fusarium oxysporum f. sp. conglutinans] | FOCO1-KAG7002927.1 |
| RKK62641.1 hypothetical protein BFJ69_g17002 [Fusarium oxysporum] | FOHP-RKK62641.1 |
| RKK62984.1 hypothetical protein BFJ69_g16960 [Fusarium oxysporum] | FOHP-RKK62984.1 |
| RKK90771.1 hypothetical protein BFJ68_g16366 [Fusarium oxysporum] | FOHP-RKK90771.1 |
| RKL21281.1 hypothetical protein BFJ68_g2549 [Fusarium oxysporum] | FOHP-RKL21281.1 |
| EWZ29335.1 hypothetical protein FOZG_16964 [Fusarium oxysporum Fo47] | FOFHP/FOHPF-EWZ29335.1 |
| QKD57451.1 hypothetical protein FOBC_11589 [Fusarium oxysporum Fo47] | FOFHP/FOHPF-QKD57451.1 |
| EGU75234.1 hypothetical protein FOXB_14280 [Fusarium oxysporum f. sp. conglutinans Fo5176] | FOCHP/FOHPC-EGU75234.1 |
| EGU78064.1 hypothetical protein FOXB_11408 [Fusarium oxysporum f. sp. conglutinans Fo5176] | FOCHP/FOHPC-EGU78064.1 |
| EGU79527.1 hypothetical protein FOXB_09961, partial [Fusarium oxysporum f. sp. conglutinans Fo5176] | FOCHP/FOHPC-EGU79527.1 |
| KAF6515179.1 hypothetical protein HZS61_005085 [Fusarium oxysporum f. sp. conglutinans] | FOCHP/FOHPC-KAF6515179.1 |
| KAF6524808.1 hypothetical protein HZS61_010603 [Fusarium oxysporum f. sp. conglutinans] | FOCHP/FOHPC-KAF6524808.1 |
