# Supplementary figures and images for: Genome-wide identification of the opsin protein in Leptosphaeria maculans and comparison with other fungi (pathogens of Brassica napus)
Source: Front Microbiol. 2023 Aug 25;14:1193892. doi: 10.3389/fmicb.2023.1193892 (PMC10485269; doi:10.3389/fmicb.2023.1193892)

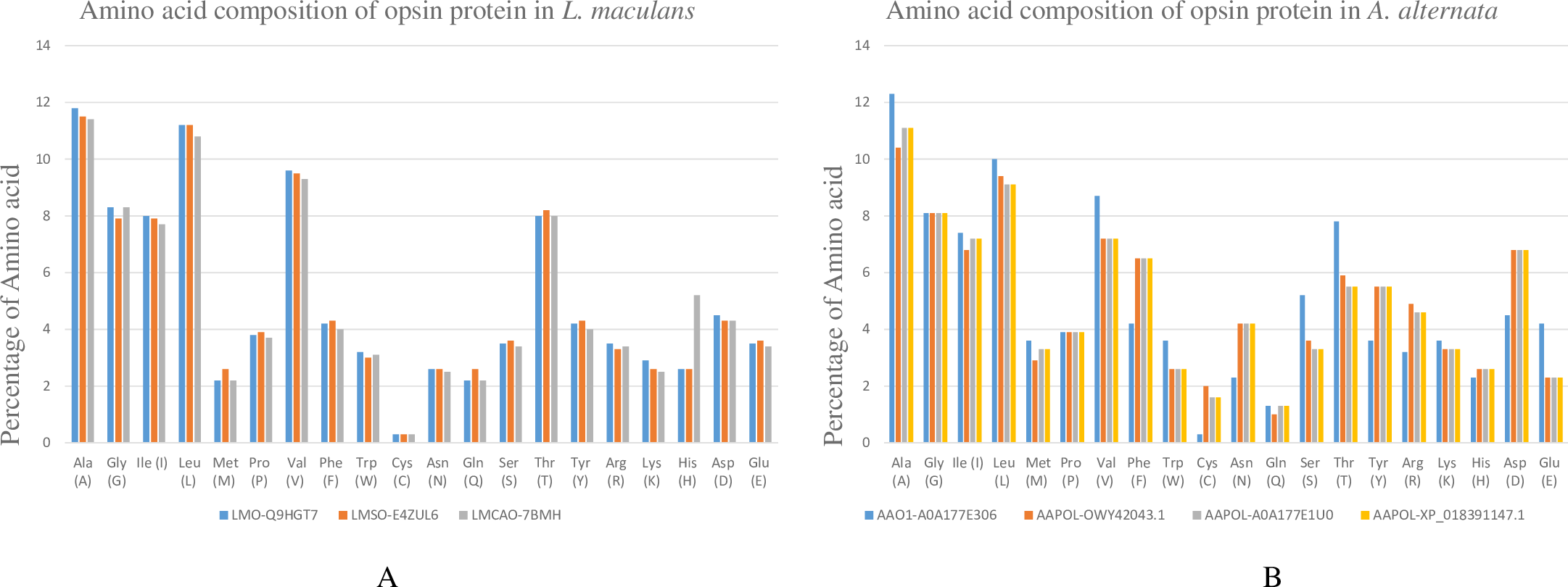

Supplement: Supplementary Figure 1 — Comparison of the amino acid composition of opsin protein in all isoforms; (A) L. maculans, (B) A. alternata, (C) S. sclerotiorum, (D) B. cinerea, (E) V. dahliae, (F) V. longisporum, (G–I) F. oxysporum. The y-axis shows the percentage of amino acids of opsin protein in each isoform; the X-axis presents the name of all amino acids. [file Data_Sheet_1.zip › Supplementary/Supplementary Figure 1 AB.tif]

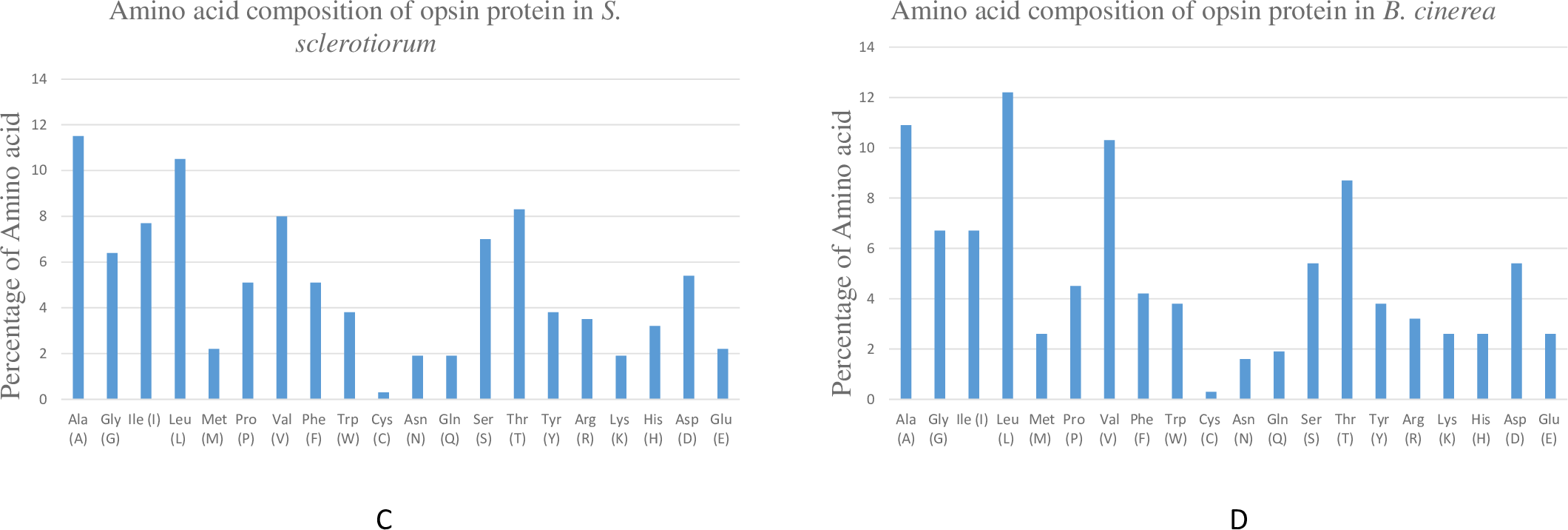

Supplement: Supplementary Figure 1 — Comparison of the amino acid composition of opsin protein in all isoforms; (A) L. maculans, (B) A. alternata, (C) S. sclerotiorum, (D) B. cinerea, (E) V. dahliae, (F) V. longisporum, (G–I) F. oxysporum. The y-axis shows the percentage of amino acids of opsin protein in each isoform; the X-axis presents the name of all amino acids. [file Data_Sheet_1.zip › Supplementary/Supplementary Figure 1 CD.tif]

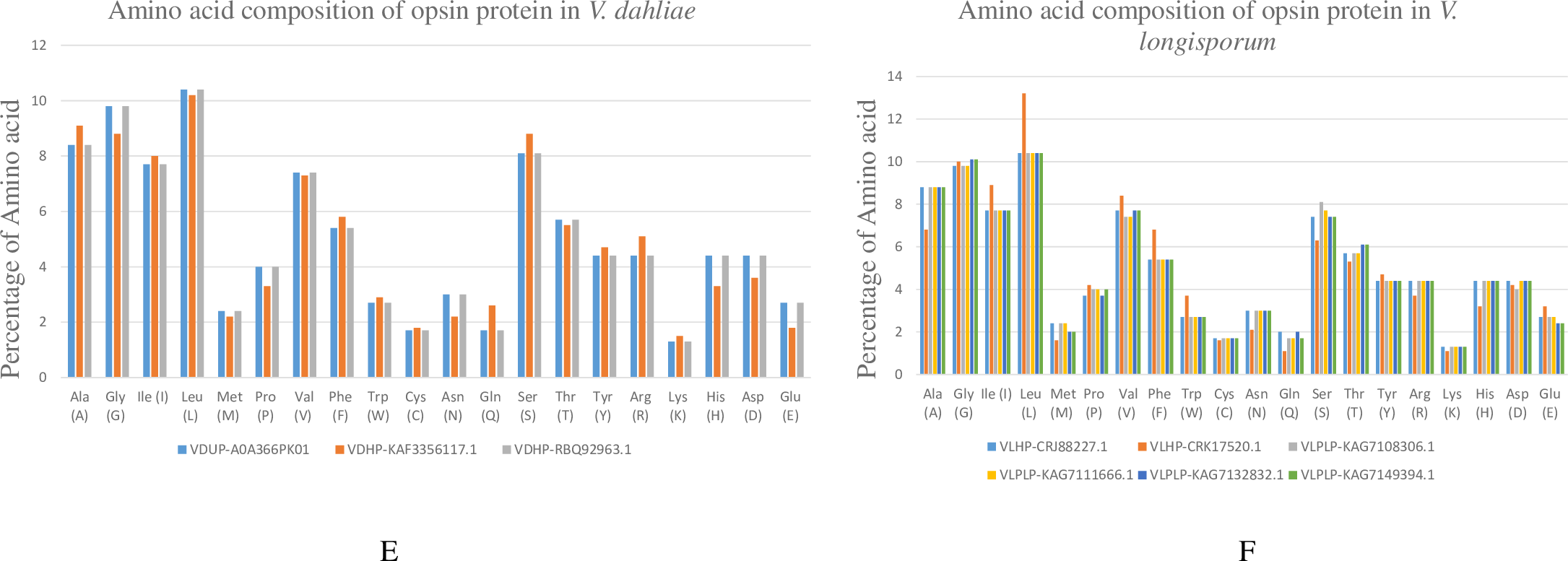

Supplement: Supplementary Figure 1 — Comparison of the amino acid composition of opsin protein in all isoforms; (A) L. maculans, (B) A. alternata, (C) S. sclerotiorum, (D) B. cinerea, (E) V. dahliae, (F) V. longisporum, (G–I) F. oxysporum. The y-axis shows the percentage of amino acids of opsin protein in each isoform; the X-axis presents the name of all amino acids. [file Data_Sheet_1.zip › Supplementary/Supplementary Figure 1 EF.tif]

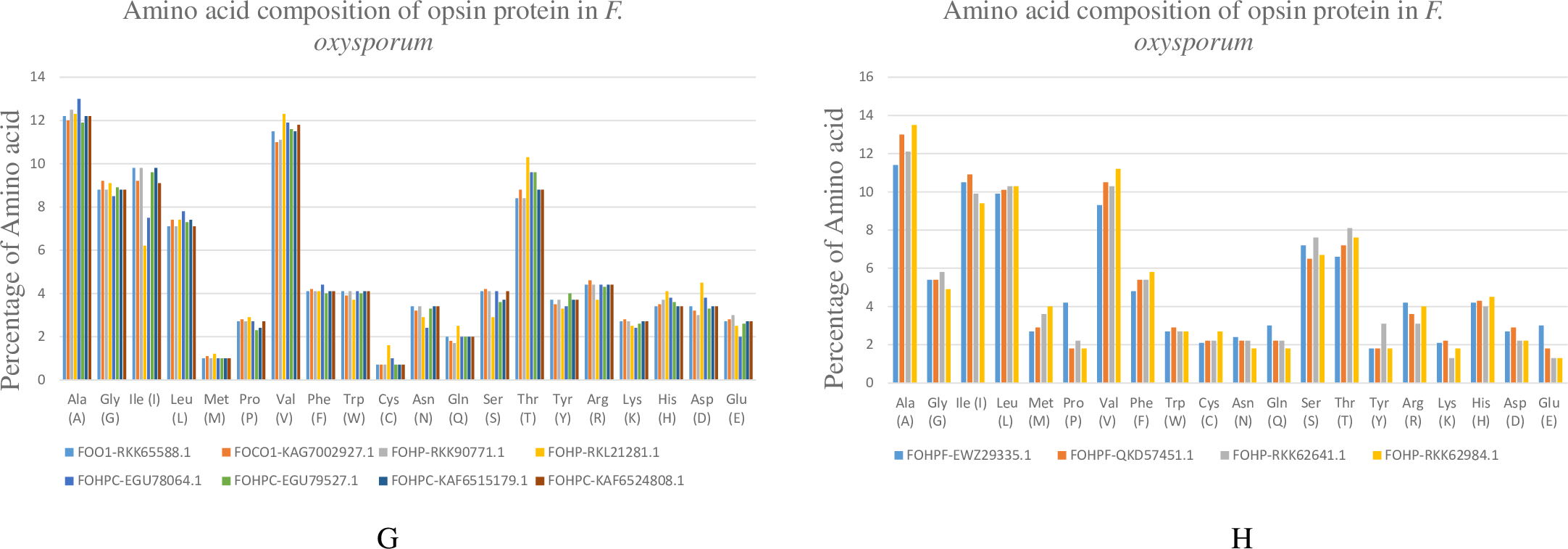

Supplement: Supplementary Figure 1 — Comparison of the amino acid composition of opsin protein in all isoforms; (A) L. maculans, (B) A. alternata, (C) S. sclerotiorum, (D) B. cinerea, (E) V. dahliae, (F) V. longisporum, (G–I) F. oxysporum. The y-axis shows the percentage of amino acids of opsin protein in each isoform; the X-axis presents the name of all amino acids. [file Data_Sheet_1.zip › Supplementary/Supplementary Figure 1 GH.tif]

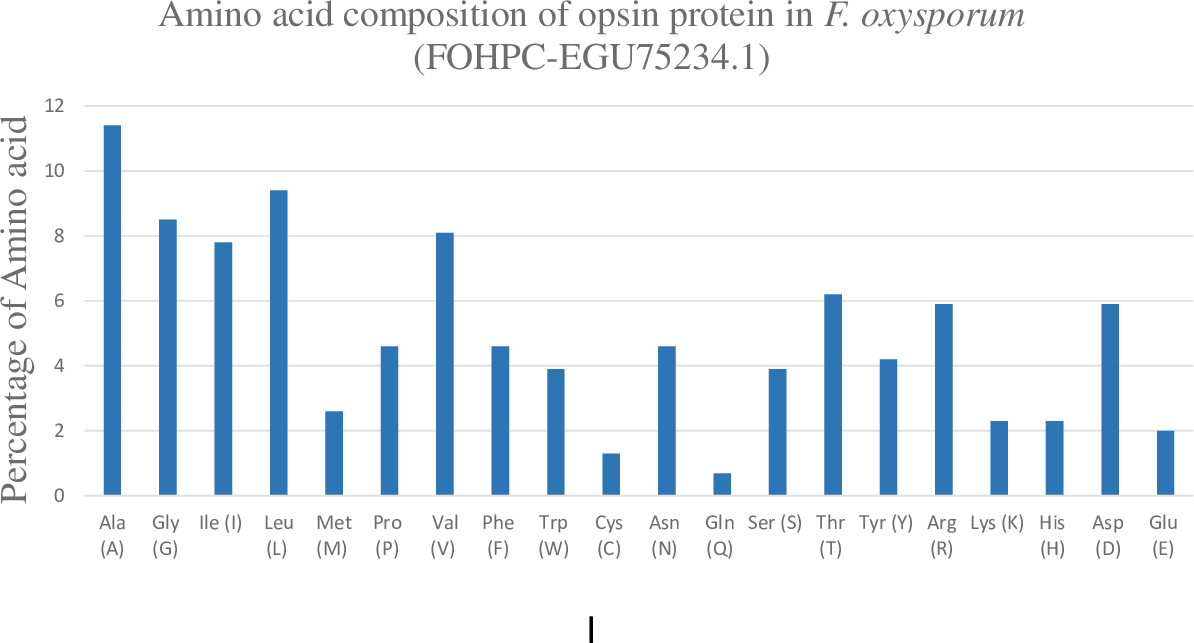

Supplement: Supplementary Figure 1 — Comparison of the amino acid composition of opsin protein in all isoforms; (A) L. maculans, (B) A. alternata, (C) S. sclerotiorum, (D) B. cinerea, (E) V. dahliae, (F) V. longisporum, (G–I) F. oxysporum. The y-axis shows the percentage of amino acids of opsin protein in each isoform; the X-axis presents the name of all amino acids. [file Data_Sheet_1.zip › Supplementary/Supplementary Figure 1 I.tif]

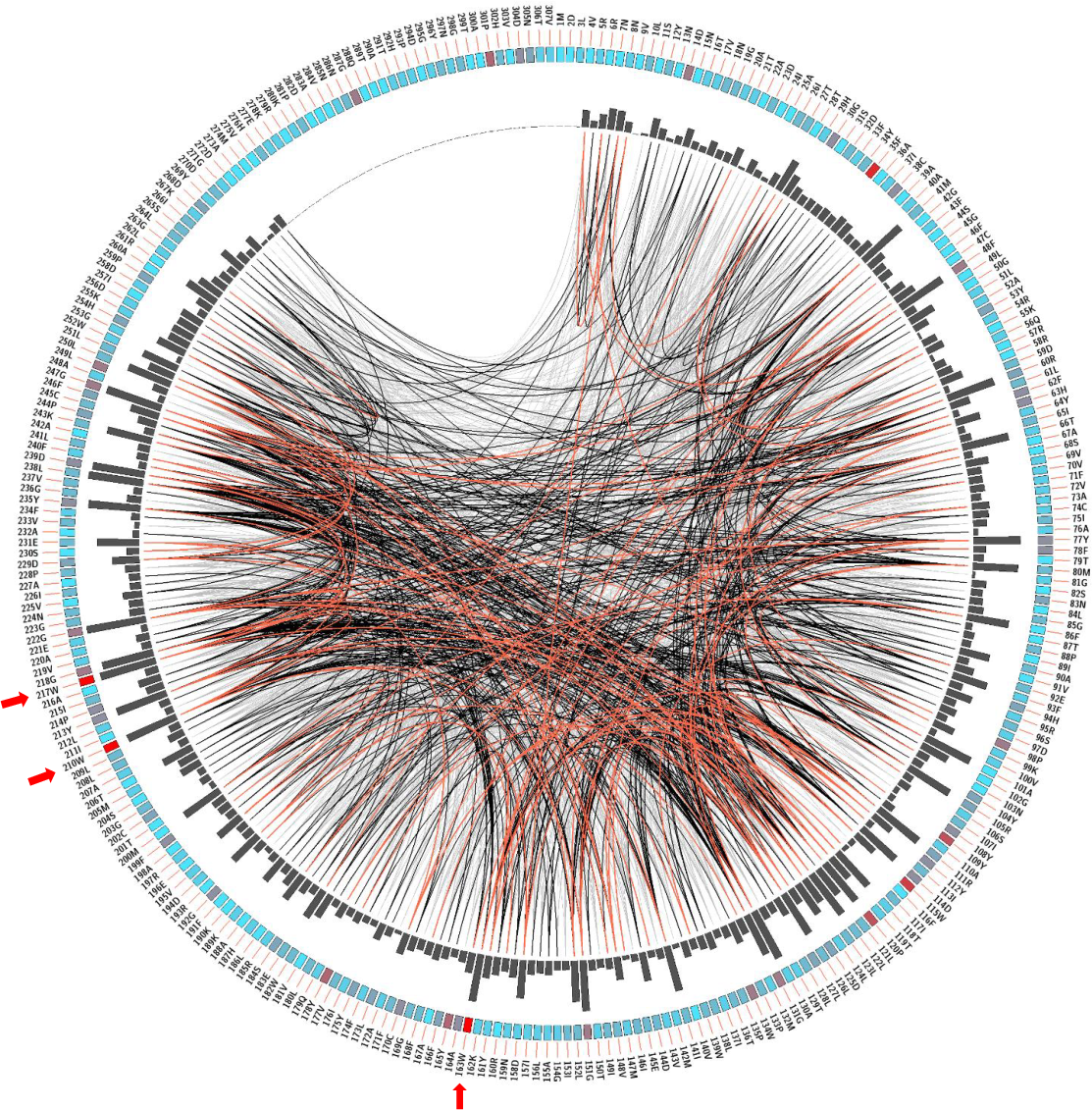

Supplement: Supplementary Figure 1 — Comparison of the amino acid composition of opsin protein in all isoforms; (A) L. maculans, (B) A. alternata, (C) S. sclerotiorum, (D) B. cinerea, (E) V. dahliae, (F) V. longisporum, (G–I) F. oxysporum. The y-axis shows the percentage of amino acids of opsin protein in each isoform; the X-axis presents the name of all amino acids. [file Data_Sheet_1.zip › Supplementary/Supplementary Figure 2.tif]

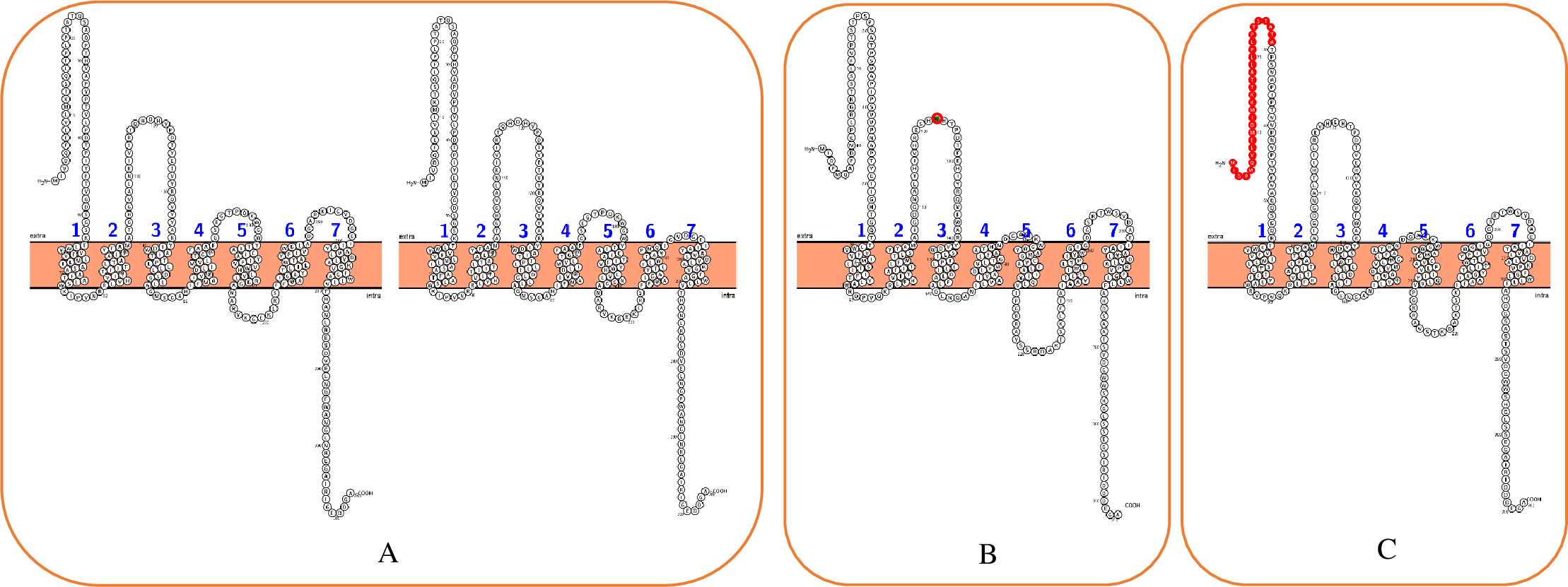

Supplement: Supplementary Figure 1 — Comparison of the amino acid composition of opsin protein in all isoforms; (A) L. maculans, (B) A. alternata, (C) S. sclerotiorum, (D) B. cinerea, (E) V. dahliae, (F) V. longisporum, (G–I) F. oxysporum. The y-axis shows the percentage of amino acids of opsin protein in each isoform; the X-axis presents the name of all amino acids. [file Data_Sheet_1.zip › Supplementary/Supplementary Figure 3 ABC.tif]

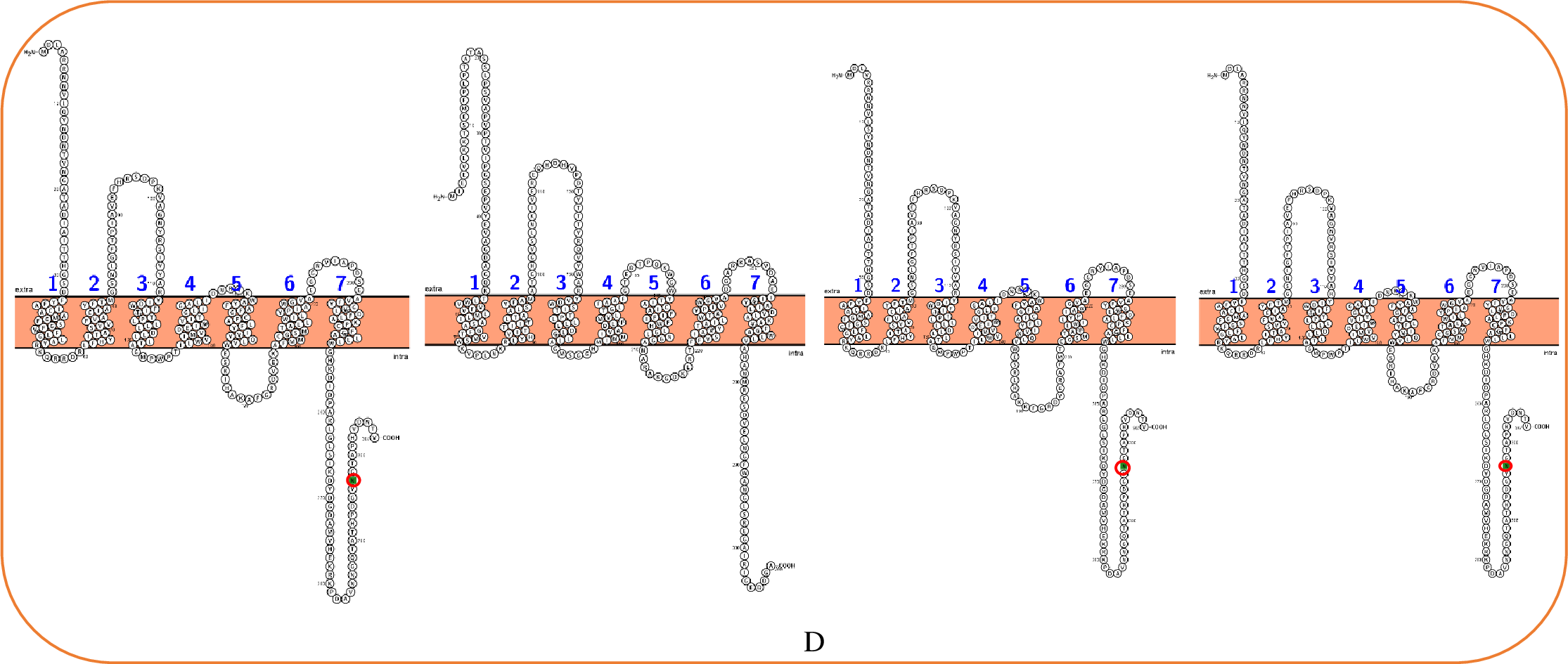

Supplement: Supplementary Figure 1 — Comparison of the amino acid composition of opsin protein in all isoforms; (A) L. maculans, (B) A. alternata, (C) S. sclerotiorum, (D) B. cinerea, (E) V. dahliae, (F) V. longisporum, (G–I) F. oxysporum. The y-axis shows the percentage of amino acids of opsin protein in each isoform; the X-axis presents the name of all amino acids. [file Data_Sheet_1.zip › Supplementary/Supplementary Figure 3 D.tif]

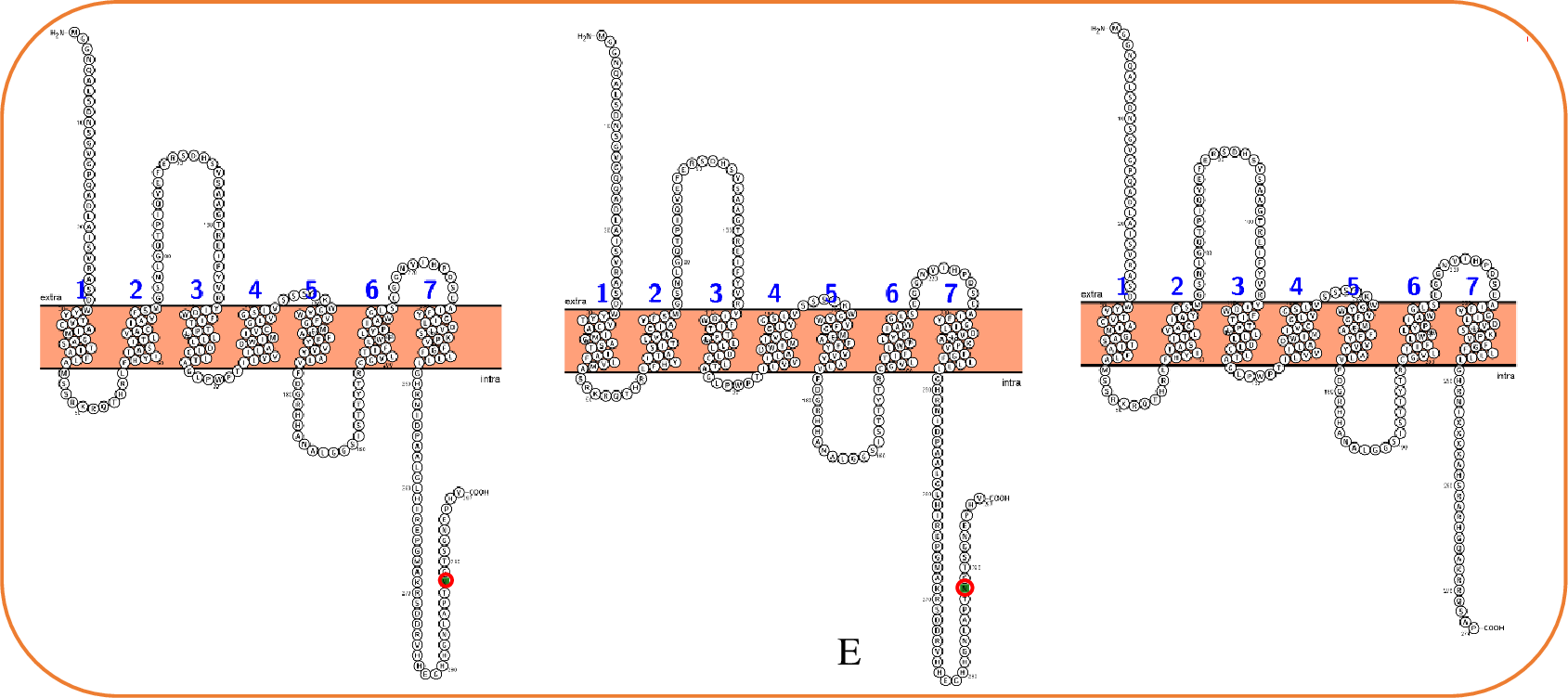

Supplement: Supplementary Figure 1 — Comparison of the amino acid composition of opsin protein in all isoforms; (A) L. maculans, (B) A. alternata, (C) S. sclerotiorum, (D) B. cinerea, (E) V. dahliae, (F) V. longisporum, (G–I) F. oxysporum. The y-axis shows the percentage of amino acids of opsin protein in each isoform; the X-axis presents the name of all amino acids. [file Data_Sheet_1.zip › Supplementary/Supplementary Figure 3 E.tif]

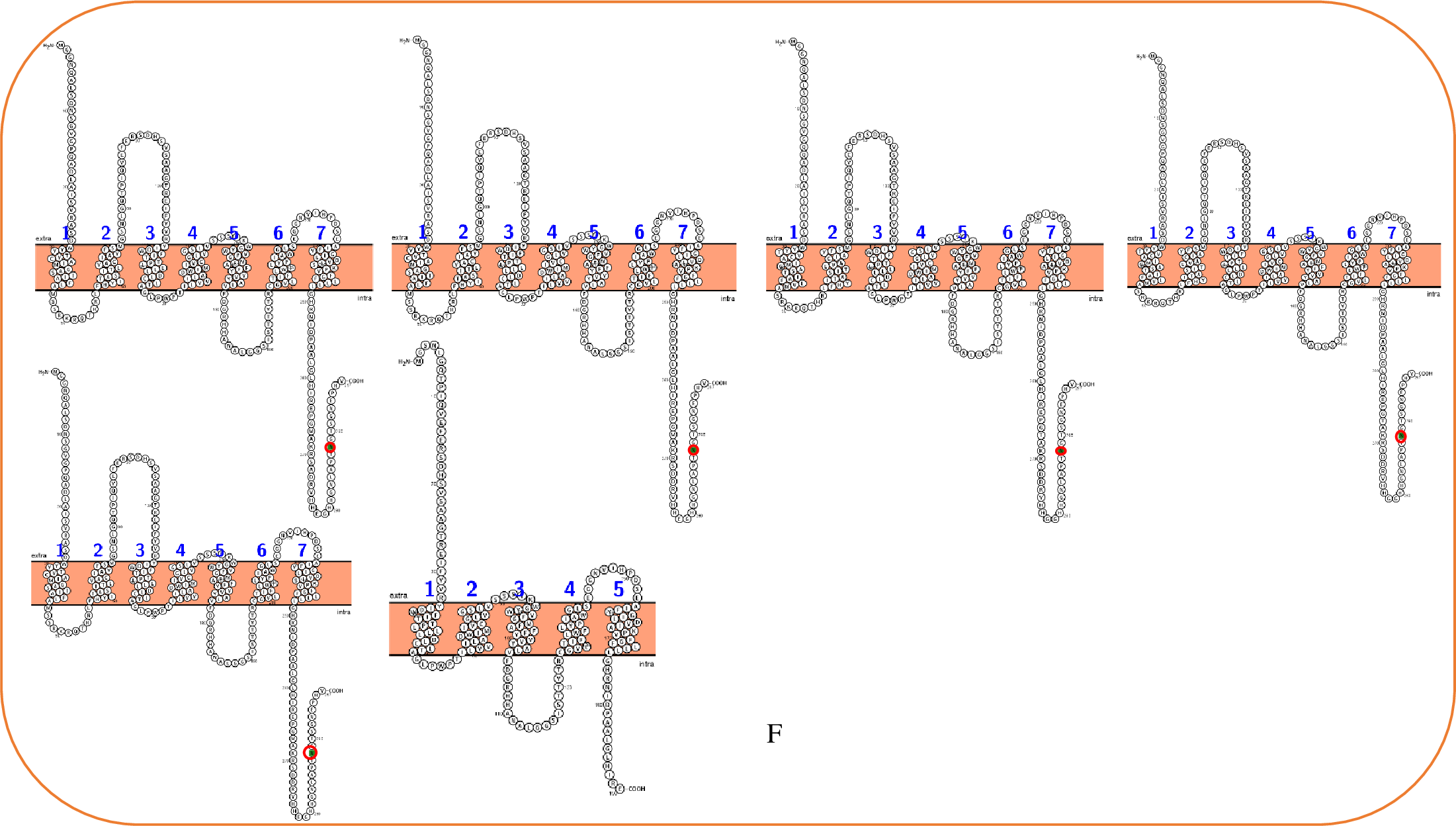

Supplement: Supplementary Figure 1 — Comparison of the amino acid composition of opsin protein in all isoforms; (A) L. maculans, (B) A. alternata, (C) S. sclerotiorum, (D) B. cinerea, (E) V. dahliae, (F) V. longisporum, (G–I) F. oxysporum. The y-axis shows the percentage of amino acids of opsin protein in each isoform; the X-axis presents the name of all amino acids. [file Data_Sheet_1.zip › Supplementary/Supplementary Figure 3 F.tif]

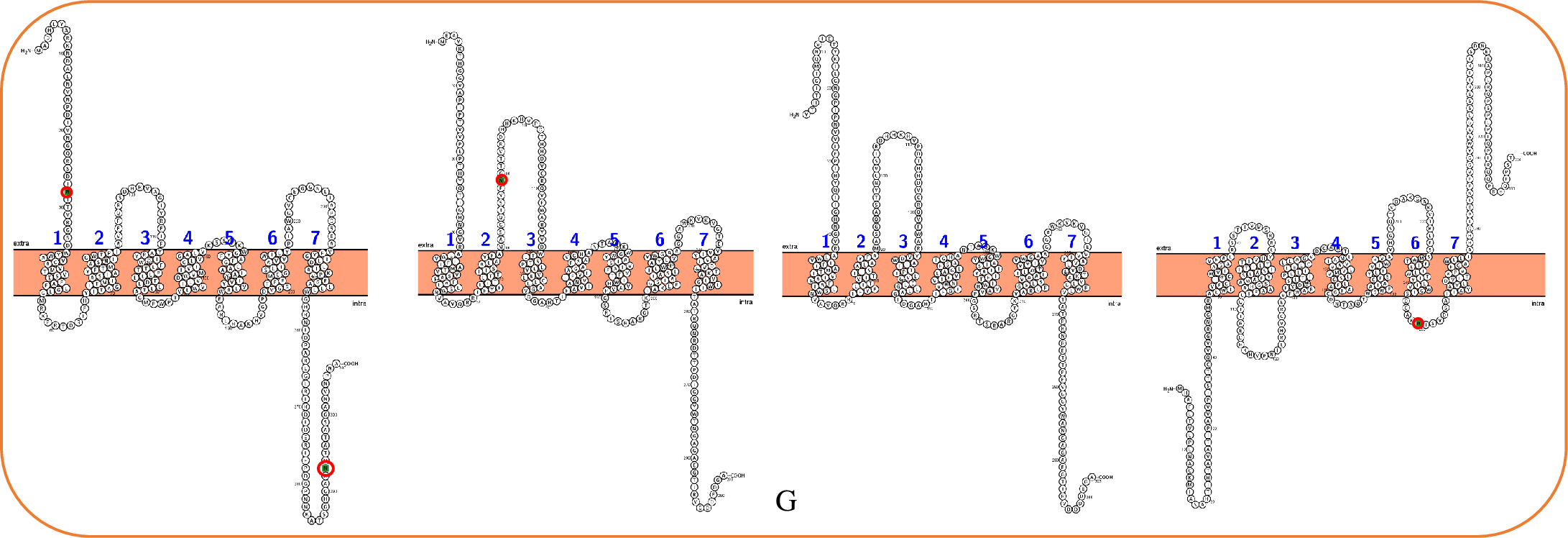

Supplement: Supplementary Figure 1 — Comparison of the amino acid composition of opsin protein in all isoforms; (A) L. maculans, (B) A. alternata, (C) S. sclerotiorum, (D) B. cinerea, (E) V. dahliae, (F) V. longisporum, (G–I) F. oxysporum. The y-axis shows the percentage of amino acids of opsin protein in each isoform; the X-axis presents the name of all amino acids. [file Data_Sheet_1.zip › Supplementary/Supplementary Figure 3 G1.tif]

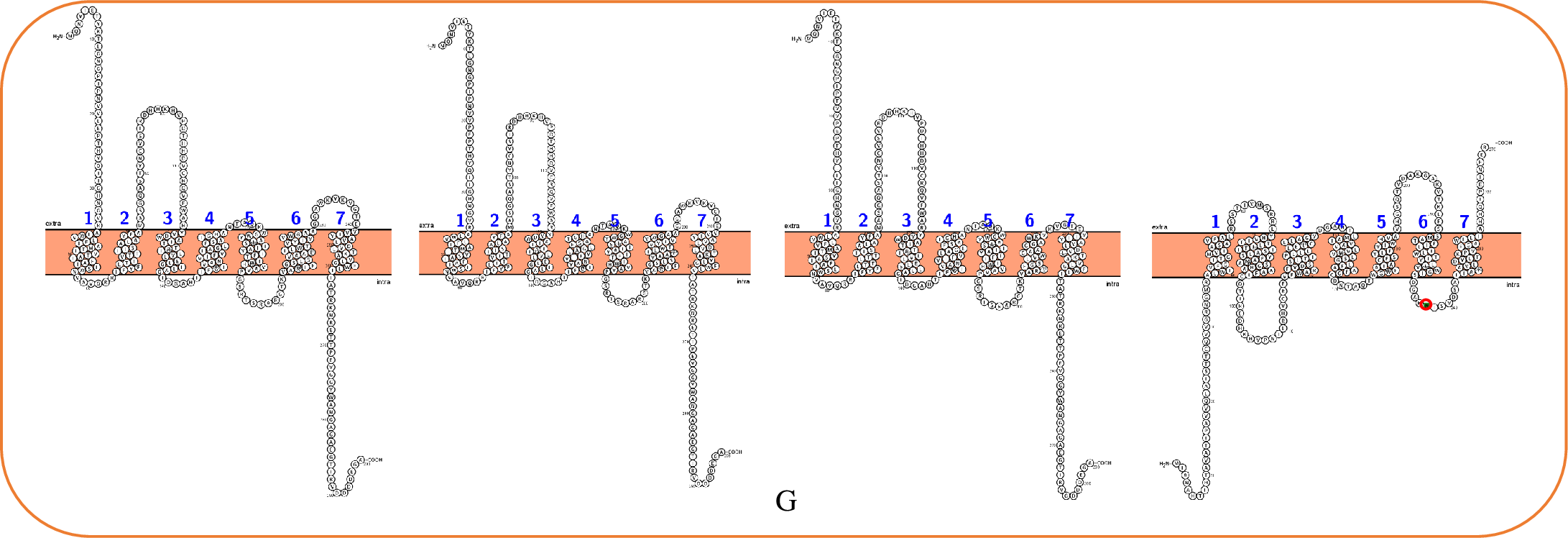

Supplement: Supplementary Figure 1 — Comparison of the amino acid composition of opsin protein in all isoforms; (A) L. maculans, (B) A. alternata, (C) S. sclerotiorum, (D) B. cinerea, (E) V. dahliae, (F) V. longisporum, (G–I) F. oxysporum. The y-axis shows the percentage of amino acids of opsin protein in each isoform; the X-axis presents the name of all amino acids. [file Data_Sheet_1.zip › Supplementary/Supplementary Figure 3 G2.tif]

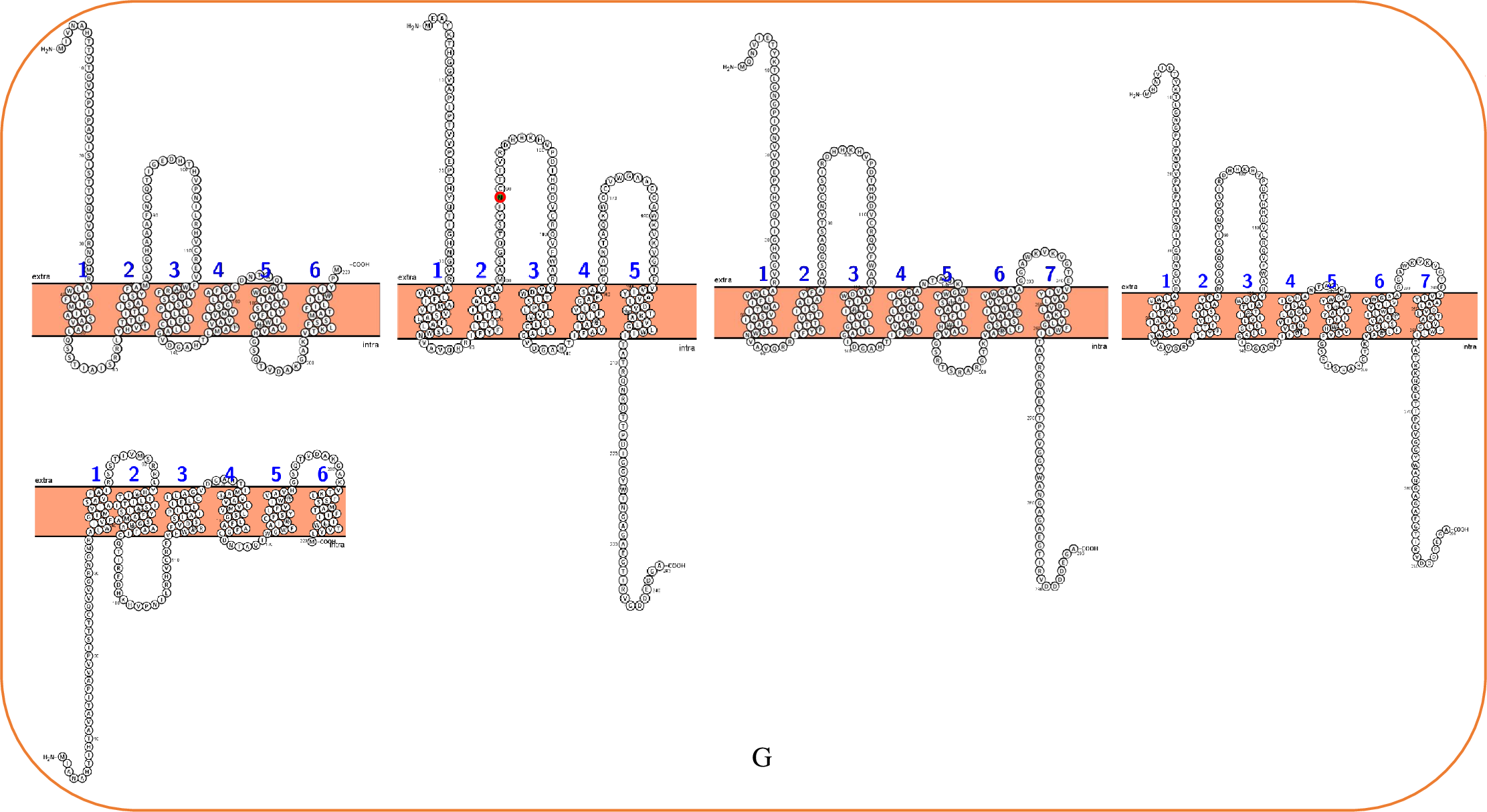

Supplement: Supplementary Figure 1 — Comparison of the amino acid composition of opsin protein in all isoforms; (A) L. maculans, (B) A. alternata, (C) S. sclerotiorum, (D) B. cinerea, (E) V. dahliae, (F) V. longisporum, (G–I) F. oxysporum. The y-axis shows the percentage of amino acids of opsin protein in each isoform; the X-axis presents the name of all amino acids. [file Data_Sheet_1.zip › Supplementary/Supplementary Figure 3 G3.tif]

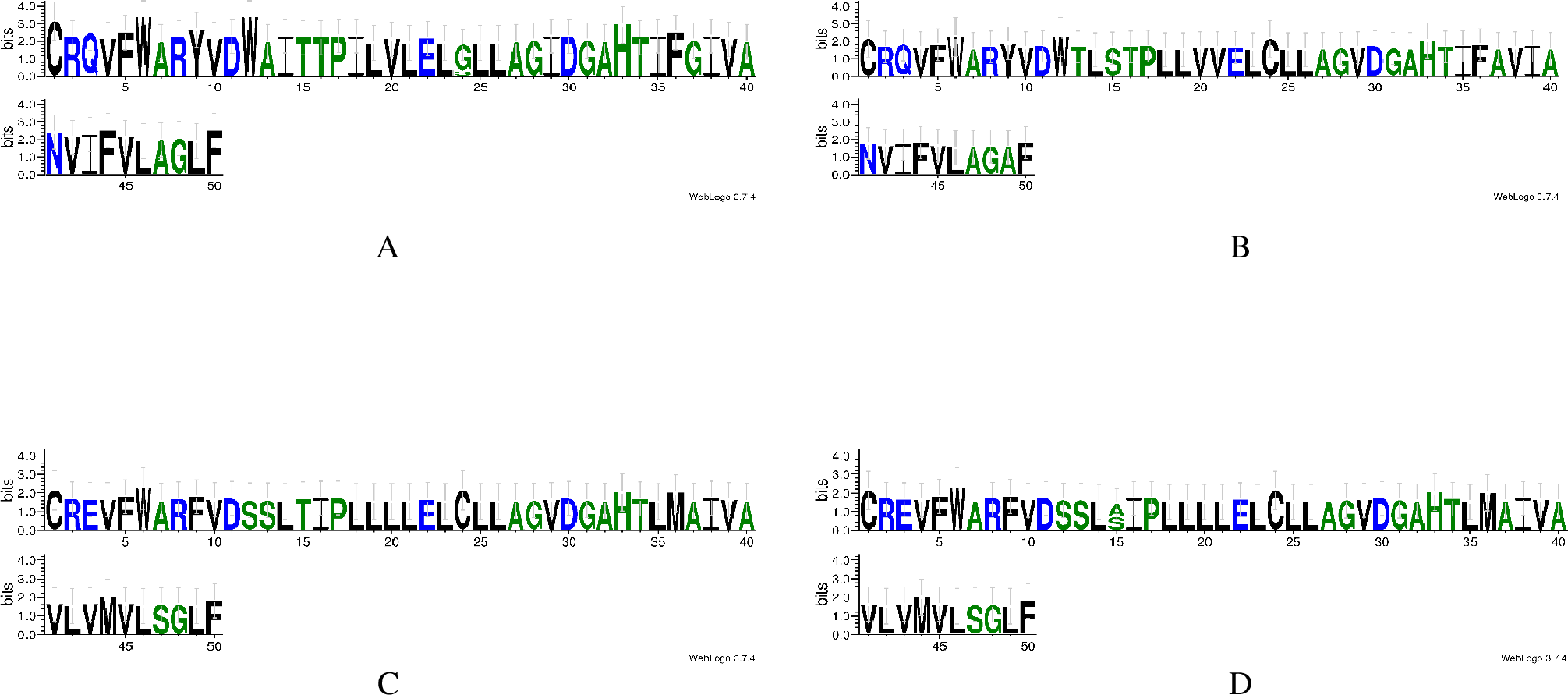

Supplement: Supplementary Figure 1 — Comparison of the amino acid composition of opsin protein in all isoforms; (A) L. maculans, (B) A. alternata, (C) S. sclerotiorum, (D) B. cinerea, (E) V. dahliae, (F) V. longisporum, (G–I) F. oxysporum. The y-axis shows the percentage of amino acids of opsin protein in each isoform; the X-axis presents the name of all amino acids. [file Data_Sheet_1.zip › Supplementary/Supplementary Figure 4 A-D.tif]

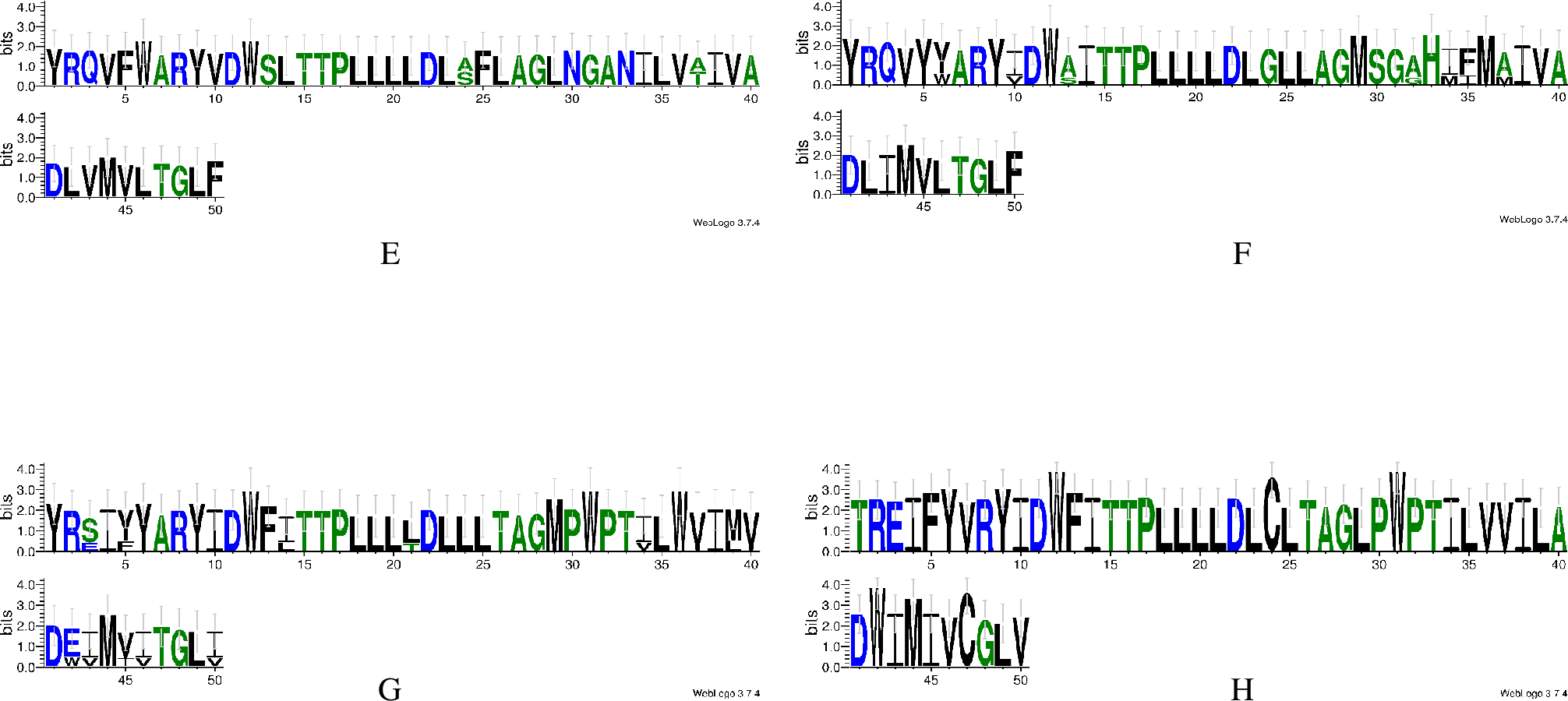

Supplement: Supplementary Figure 1 — Comparison of the amino acid composition of opsin protein in all isoforms; (A) L. maculans, (B) A. alternata, (C) S. sclerotiorum, (D) B. cinerea, (E) V. dahliae, (F) V. longisporum, (G–I) F. oxysporum. The y-axis shows the percentage of amino acids of opsin protein in each isoform; the X-axis presents the name of all amino acids. [file Data_Sheet_1.zip › Supplementary/Supplementary Figure 4 E-H.tif]

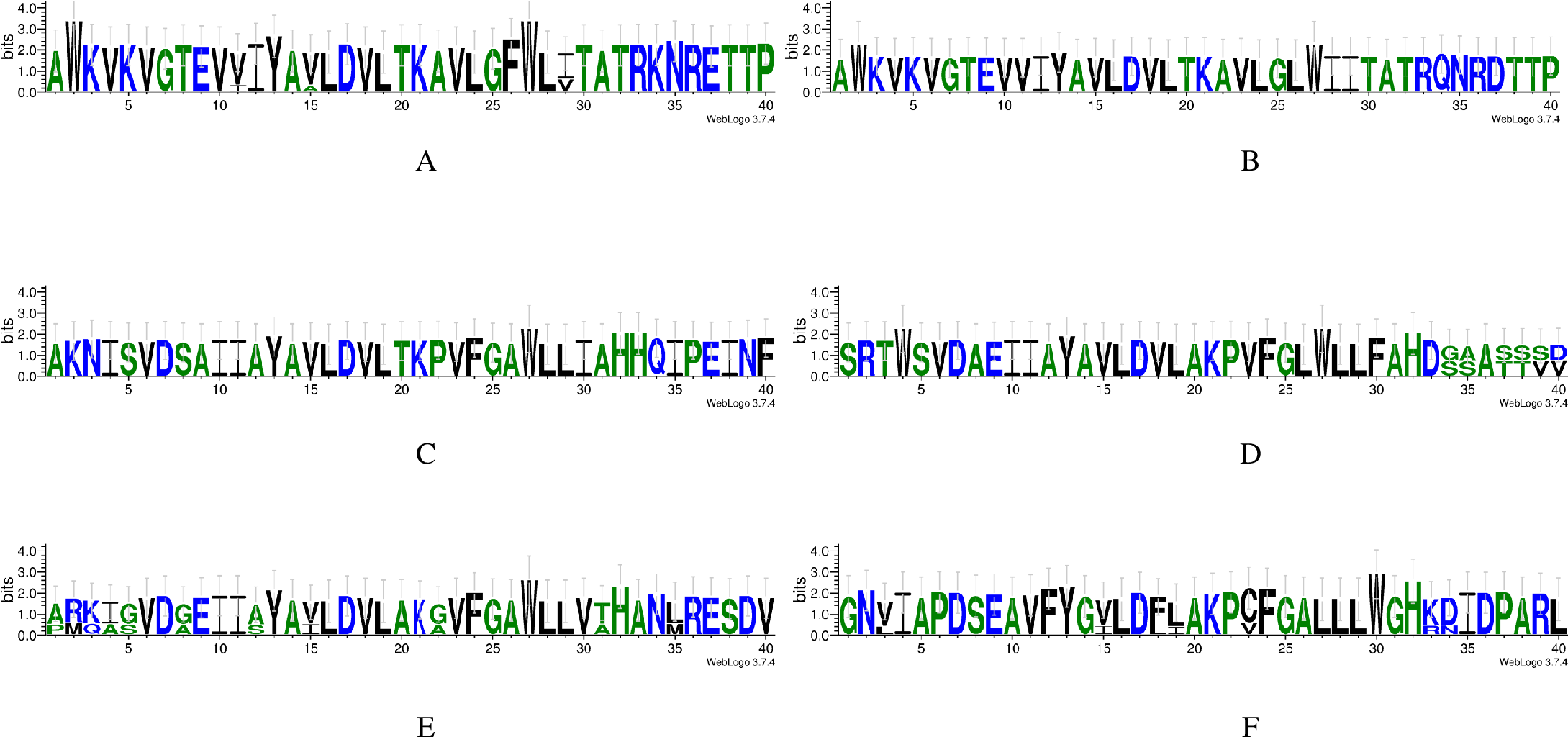

Supplement: Supplementary Figure 1 — Comparison of the amino acid composition of opsin protein in all isoforms; (A) L. maculans, (B) A. alternata, (C) S. sclerotiorum, (D) B. cinerea, (E) V. dahliae, (F) V. longisporum, (G–I) F. oxysporum. The y-axis shows the percentage of amino acids of opsin protein in each isoform; the X-axis presents the name of all amino acids. [file Data_Sheet_1.zip › Supplementary/Supplementary Figure 5 A-F.tif]

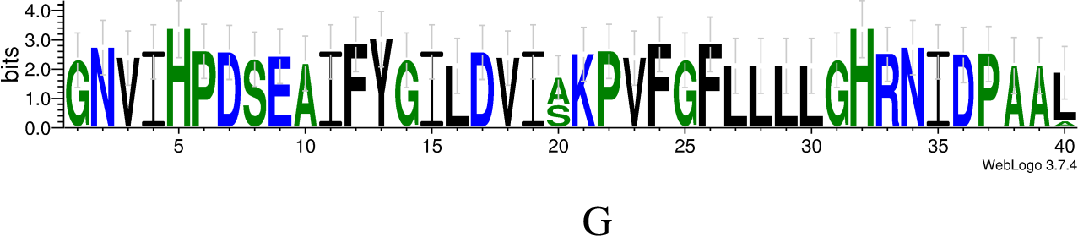

Supplement: Supplementary Figure 1 — Comparison of the amino acid composition of opsin protein in all isoforms; (A) L. maculans, (B) A. alternata, (C) S. sclerotiorum, (D) B. cinerea, (E) V. dahliae, (F) V. longisporum, (G–I) F. oxysporum. The y-axis shows the percentage of amino acids of opsin protein in each isoform; the X-axis presents the name of all amino acids. [file Data_Sheet_1.zip › Supplementary/Supplementary Figure 5 G.tif]

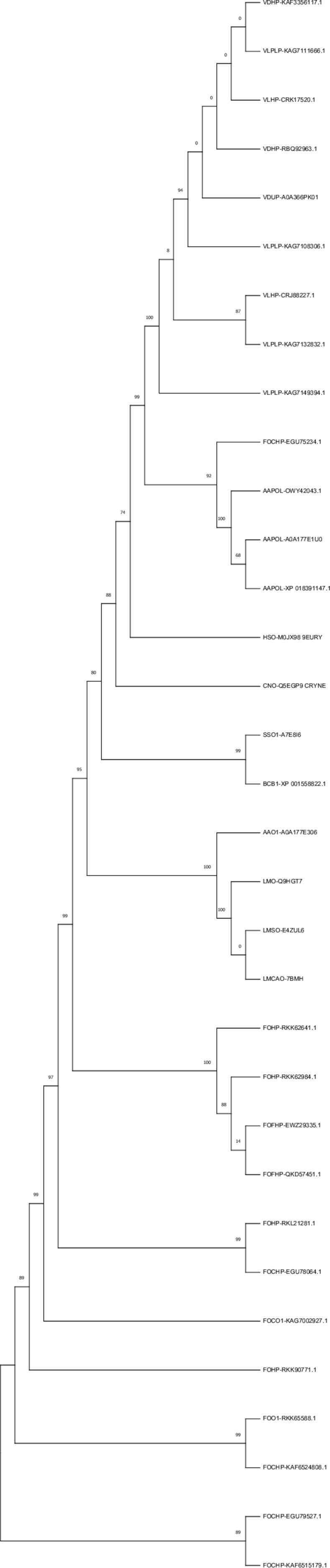

Supplement: Supplementary Figure 1 — Comparison of the amino acid composition of opsin protein in all isoforms; (A) L. maculans, (B) A. alternata, (C) S. sclerotiorum, (D) B. cinerea, (E) V. dahliae, (F) V. longisporum, (G–I) F. oxysporum. The y-axis shows the percentage of amino acids of opsin protein in each isoform; the X-axis presents the name of all amino acids. [file Data_Sheet_1.zip › Supplementary/Supplementary Figure 6.tif]
